# Uncovering Major Structural and Functional Features of Methyl-Coenzyme M Reductase (MCR) from *Methanobrevibacter ruminantium* in Complex with Two Substrates

**DOI:** 10.3390/ijms27020995

**Published:** 2026-01-19

**Authors:** Han-Ha Chai, Woncheoul Park, Dajeong Lim

**Affiliations:** 1 Precision Animal Nutrition Division, National Institute of Animal Science, Rural Development Administration, Wanju 55365, Republic of Korea; 2 Animal Breeding & Genetics Division, National Institute of Animal Science, Rural Development Administration, Wanju 55365, Republic of Korea; 3 Department of Animal Resources Science, College of Agriculture and Life Sciences, Chungnam National University, Daejeon 34134, Republic of Korea

**Keywords:** methyl-coenzyme M reductase, *Methanobrevibacter ruminantium*, enteric methane, methane mitigation, homology modeling, structure-based pharmacophore, molecular docking

## Abstract

Structural insights into methyl-coenzyme M reductase from *Methanobrevibacter ruminantium* (*M. ruminantium*) has implications for methane mitigation strategies. Methanogenesis in ruminants is a major contributor to global greenhouse gas emissions, primarily driven by the rumen archaeon *M. ruminantium*. Central to this process is methyl-coenzyme M reductase (Mcr), an enzyme that catalyzes the final step of methane production. Despite its significance as a chemogenetic target for methane mitigation, the high-resolution structure of *M. ruminantium* Mcr has remained elusive. Here, we employed homology modeling and CDOCKER simulations within the CHARMM force field to elucidate the structural and functional features of the *M. ruminantium* Mcr/ligand complexes. We characterized two distinct states: the reduced Mcr_oxi-silent_ state bound to HS-CoM and CoB-SH, and the oxidized Mcr_silent_ state bound to the heterodisulfide CoM-S-S-CoB. Alanine-scanning mutagenesis identified 71 and 62 key residues per active site for each state, respectively, revealing the fundamental determinants of structural stability and substrate selectivity on the Ni-F_430_ cofactor. Furthermore, structure-based pharmacophore modeling defined essential features (AAADDNNN and AAADDNN) that drive ligand binding. These findings provide a high-resolution molecular framework for the rational design of specific Mcr inhibitors, offering a robust starting point for developing broad-spectrum strategies to suppress enteric methane emissions.

## 1. Introduction

Ruminants including cattle, sheep, and goats are evolutionarily distinguished by a specialized forestomach system, where the rumen serve as a paramount bioreactor. This organ facilitates enteric fermentation, covering low-quality lignocelluosic biomass into high-quality nutrients that provide up to 70% of the host’s daily energy requirements via volatile fatty acids (VFAs) [1,2,3]. However, this vital metabolic process is intrinsically coupled with the production of methane (CH_4_), a byproduct of anaerobic microbial ecosystem. Rumen methanogens, dominated by the phylum *Euryarchaeota* (10^6^ to 10^8^ cells/mL), represent a critical yet environmentally problematic niche within the microbiota [4,5]. Recent large-scale genomic initiatives, such as the Hungate 1000 project, underscore the prevalence of these archaea, revealing that nearly 90% of the archaeal community is composed of just five genera, with *Methanobrevibacter* (63.2%) being the most dominant [6].

Within the rumen’s trophic chain, methanogenic function as terminal electron scavengers, primarily via the hydrogenotrophic pathway (78%), which utilizes H_2_ and CO_2_ derived from bacterial fermentation [7]. While this process maintains low H_2_ partial pressure and promotes efficient cellulolytic digestion, it imposes a significant bioenergetics tax on the host, consuming 2% to 12% of gross energy intake [8] (Figure S1). Beyond the loss of animal productivity, enteric CH_4_ is a potent greenhouse gas with a global warming potential 28 times that of CO_2_, positioning methanogenic archaea as a primary target for global climate change mitigation [9,10,11].

Despite extensive efforts to inhibit methanogenesis through various chemical and enzymatic interventions, achieving high efficacy without disrupting the boarder rumen fermentation balance remains a challenge [12,13]. Many existing anti-methanogenic compounds suffer from low specificity, potential host toxicity, and the rapid emergence of microbial resistance, often leading to methanogen recolonization. Consequently, there is an urgent need to identify highly conserved, methanogen-specific molecular targets [14]. A precision chemogenomic approach that targets essential metabolic enzymes minimizing effective concentrations while maximizing specificity offers a promising frontier for sustainable methane mitigation. In the study, we elucidate the structural and functional determinants of the methanogenesis pathway in *Methanobrevibacter ruminantium,* proving a molecular framework for the rational design of next-generation, specific inhibitors [15,16].

To effectively mitigate enteric methane, chemical interventions must selectively disrupt the metabolic activity of methanogens without perturbing the broader rumen microbiome. Among potential targets, the hydrogenotrophic methanogenesis pathway, the predominant route for CH_4_ production in ruminants, is particularly significant. High methane-emitting bovines exhibit a distinct shift in the rumen community toward lower hydrogenotrophic diversity and a compensatory increase in the abundance of *M. ruminantium*. Genome-wide analysis of *M. ruminantium* (e.g., strain M1, 2.93 Mb) reveals a specialized enzymatic repertoire dedicated to converting CO_2_, formate, and methyl-groups into CH_4_ [7,17,18,19] as shown in Figure S2. This metabolic obligacy, coupled with the genus’s high representation (63–68% of total rumen archaea) [20,21,22], underscores *M. ruminantium* as a primary focal point for chemogenetic strategies.

At the heart of this pathway lies methyl-coenzyme M reductase (Mcr), the definite enzyme for methanogenic energy metabolism (Figure S3). Mcr catalyzes the reversible reduction of methyl-coenzyme M (CH_3_-S-CoM) with coenzyme B (CoB-SH) to yield CH_4_ and the heterodisulfide CoM-S-S-CoB [23,24]. This terminal step is highly efficient, with a catalytic forward reaction rate exceeding the reverse methane oxidation by five orders of magnitude. Structural specificity is paramount, for instance, the enzyme exhibits a 1000-fold preference for its native C1 substrate over larger analogs like ethyl-coenzyme M, due to steric constraints within its 30 Å-long narrow hydrophobic channel [25,26,27].

Crucially, the catalytic competence of Mcr is strictly dependent on the Ni-F_430_ cofactor, a unique nickel-containing tetrapyrrole. The enzyme is active only when the nickel center is in the highly reduced Ni(I) state, making it sensitive to the redox environment and growth-rate limiting under substrate poor conditions. Despite the availability of high-resolution structures of other methanogens, the specific molecular architecture of *M. ruminantium* Mcr has not been empirically determined. Given that energy metabolism is the primary driver of methanogen growth, targeting the Mcr active site specifically the Ni-F_430_ interaction interface offers a robust pathway to suppress CH_4_ emissions. In this study, we utilize comparative genomics and advanced structural modeling to define the chemogenetic features of *M. ruminantium* Mcr, providing a molecular basis for the development of high affinity, specific inhibitors with broad efficacy across rumen methanogens.

## 2. Results

The *M. ruminantium* encodes two-Mcr isoenzymes, Mcr I and Mcr II, which exhibit differential expression patterns depending on the growth phase and hydrogen availability. Mcr I (isoenyme I, mcr) is primarily synthesized during steady-state growth on H_2_ and CO_2_, whereas Mcr II (isoenzyme II, mtr) is upregulated under H_2_-replete conditions, particularly when H_2_ levels become limiting at high cell densities [28]. At the loci level, the expression of Mcr I and Mcr II is governed by two separate operons, mcrBGCDA and mtrBGDA, which facilitate the differential regulation of these isoenzymes [29]. In hydrogenotrophic methanogens, the amino acid sequences of Mcr I and Mcr II share 60–70% identity [30]. The functional Mcr I isoenzyme adopts an α_2_β_2_γ_2_ heterohexameric stoichiometry, comprising two states of these distinct subunits: α (McrA), β (McrB), and γ (McrG). While the accessory proteins McrC and McrD are not intrinsic to the catalytic core, they are essential for post-translational maturation process; specifically, McrD facilitates complex assembly, whereas McrC is required for enzymatic activation [28]. The Mcr I system predominates in *M. ruminantium* during growth dependent on exogenous acetate, 2-methylbutyrate, and coenzyme M (2-mercaptoethanesulfonate) under low-H_2_ conditions. Notably, this species lacks the genomic architecture for a Mcr II system [12]. Acetate serves as a critical carbon source for biosynthesis in *M. ruminantium*, initiated by its activation to acetyl-CoA (facilitated by acs and acsA) and subsequent reductive carboxylation to pyruvate, mediated by the porABCDEFG gene cluster as shown in Figures S1 and S2. Similarly, the reductive carboxylation of 2-methylbutyrate is proposed as the primary pathway for isoleucine biosynthesis. Central to energy conservation, coenzyme M remains indispensable for the terminal methanogenic step during vegetative growth. Notably, the majority of these methanogen-specific genes particularly those governing the core energy metabolism of the methanogenesis pathways (Figures S2–S5) are highly conserved across diverse rumen methanogens [31,32,33], underscoring their evolutionary importance for survival within the competitive ruminal ecosystem.

Given that most methanogenic enzymes are localized within the cytoplasm, this compartment has emerged as a primary factor for the development of targeted inhibitors. Of particular interest is Mcr, the terminal enzyme of the methanogenesis pathway. Mcr is a 300 kDa complex structured as a C_2_-symmetric heterohexamer (α_2_β_2_γ_2_), featuring two active sites that house the essential nickel-containing F_430_ cofactor. In its fully reduced state, the Mcr enzyme exhibits specific activities ranging from 50 to 100 U/mg, a catalytic proficiency contingent upon the Ni(I) oxidation state of its F_430_ cofactor [34]. Structural analysis across 29 known crystal structures demonstrates that the Mcr complex architecture defines two distinct active sites, spatially separated by approximately 50 Å (represented in Figure S10). The catalytic center is sequestered within a 30 Å-long narrow substrate channel approximately three times the length of coenzyme B which extends from the enzyme surface to the interior of the Mcr complex. This hydrophobic conduit facilitates the precise orientation of coenzyme HS-CoM and CoB-SH, or the heterodisulfide product CoM-S-S-CoB, in immediate proximity to the Ni-containing F_430_ cofactor. The substrate channel and coenzyme binding pockets are lined by a conserved network of positively charged, non-polar, and aromatic residues. These sites are formed at the interfaces of the α_2_β_2_γ_2_ heterohexamer, notably, a single αβγ heterotrimer is functionally insufficient. Full catalytic activity requires the complete hexameric assembly, where the precise orientation of subunits from both trimeric halves specifically the reciprocal interactions between subunits α, α′, β and γ, and their symmetry-related counterparts (α′, α, β′, and γ′) constitutes the active holoenzyme. Each active site harbors a unique Ni-containing porphinoid, cofactor F_430_, embedded deep within the catalytic pocket. Notably, the enzymatic activity of Mcr is strictly proportioned to the abundance of the Ni(I) oxidation site, which serve as the requisite electronic configuration for catalysis [35,36]. Each active site is accessible to the first substrate, methyl-coenzyme M [CH_3_-S-CoM; 2-(methylthio)ethanesulfonate], with a dissociation constant (K_d_) of 17 µM. The binding of CH_3_-S-CoM induces a conformational restriction that enhances the enzyme’s affinity for its second substrate, coenzyme B (CoB-SH; 7-mercaptoheptanoylthreonine phosphate), resulting in a K_d_ of 79 µM for the Mcr-CH_3_-S-CoM/CoB-SH ternary complex [37]. Kinetic simulations further indicate that the dissociation constant for CoB-SH within the inhibitory Mcr/CoB-SH complex is K_d_ = 96 µM. Structural constraints dictate that the active site is accessible only via a single substrate channel, which imposes a strict size exclusion limit. This conduit features a funnel-like architecture, originating with a 25 Å diameter at the protein surface and tapering to 8 Å as it approaches the F_430_ cofactor at the pocket base [28]. Consequently, only small molecules with diameters up to 6 Å, such as CH_3_-S-CoM [38], can penetrate the interior to reach the catalytic center. This spatial arrangement brings the CH_3_-S-CoM substrate into close proximity to the nickel center, where the Ni(I) ion facilitates C-S bond cleavage. A compelling mechanistic model proposes that Ni(I) acts as a nucleophile in an S_N_2-type reaction with CH_3_-S-CoM and CoB-SH, potentially generating a methyl-nickel(III) intermediate (Ni(III)-CH_3_) alongside a sulfanyl radical (S-CoM) and a thioanion (CoB-S^−^) [36,39]. Furthermore, the two isoenzymes exhibit distinct kinetic profiles; whereas Mcr I shows a pH optimum of 7.0–7.5, Mcr II is characterized by a slightly more alkaline optimum between 7.5 and 8.0 [28,38].

### 2.1. Molecular Basis of Substrate Recognition in the Mcr_oxi-silent_ Complexes

**Figure 1:**
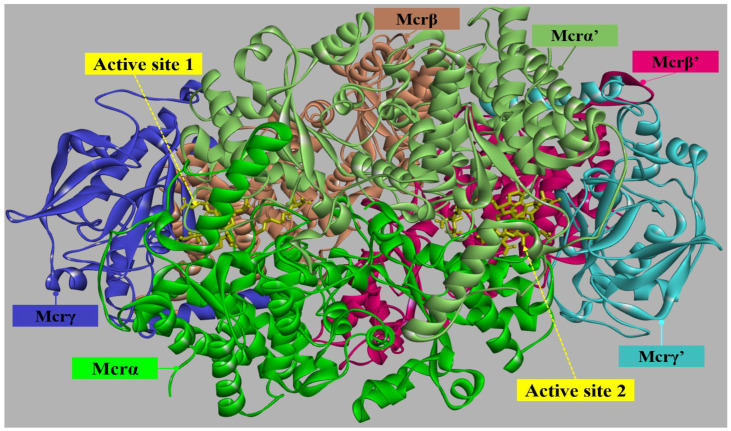
Structural organization of the *Methanobrevibacter ruminantium* Mcr (αβγ)_2_ heterohexamer. The α and α′ subunits are rendered in green, β and β′ in magenta, and γ and γ′ subunits in blue. In this multi-subunit complex, each subunit establishes extensive contact with all other structural components. The F_430_ cofactor and substrates (coenzyme M and coenzyme B) are highlighted as yellow spheres. Each active site is formed by the quaternary assembly of four subunits α, α′, β and γ (and symmetrically α′, α, β′ and γ′). As summarized in Table 1, both active sites exhibit nearly identical structural arrangements, operating independently. The F_430_ cofactor is precisely accommodated within the pocket situated at the apex of the substrate channel within each catalytic site.

**Figure 2:**
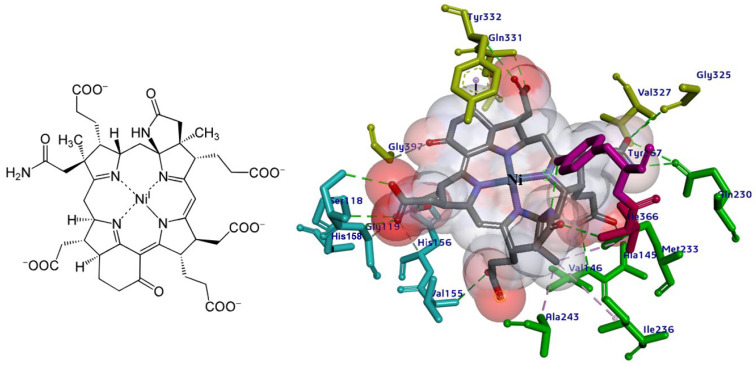
Structural organization and binding interface of the F_430_ cofactor within *M. ruminantium* Mcr active site. The F_430_ cofactor characterized by a unique titled pyrrole configuration, is rigidly anchored within the Mcr enzyme, adopting a largely planar conformation with hexacoordinate Ni^2+^. Non-covalent interactions between the F_430_ carboxylate groups and the Mcr side chains are extensive. This includes a robust H-bonding network (represented by fifteen dashed lines) involving the peptide amide nitrogens, as well as crucial hydrophobic interactions with the atomic ring system of Mcrα Tyr332 and Mcrβ Tyr367 residues, which are proximal to the cofactor. Active site 1 interface residues are color-coded by subunit: Mcrα (yellow), Mcrβ (pink), Mcrγ (blue), and Mcrα′ (green).

In the α_2_β_2_γ_2_ heterohexameric model of *M. ruminantium* Mcr, the α and β subunits exhibit strikingly similar fold topologies. Their core structures are characterized by a complex bundle of parallel helices and a four-stranded antiparallel β-sheet, which together constitute an α-β sandwich architecture that encapsulates the internal helical regions. The α and β subunits are oriented perpendicular to one another, with their interface stabilized by the four-stranded β-sheet of the γ subunit. This γ subunit adopts a characteristic α/β fold, complemented by an extended C-terminal helix and a flexible N-terminal loop, which together scaffold the heterotrimeric junction (as represented in Figures S6–S8). These structures share a conserved 3D scaffold with the αβγ heterotrimeric template (as shown in Figure 1). Upon dimerization, the reconstructed *M. ruminantium* Mcr assembly yields a 269.9 kDa α_2_β_2_γ_2_ heterohexamer. Despite the structural homology, the α and β subunits are functionally distinct with the catalytic environment. Two Ni-porphinoid F_430_ cofactors are deeply embedded at the interfaces of symmetry-related subunits specificity between α, α′, β, and γ and α′, α, β′, and γ′ clusters creating two spatially discrete yet structurally identical active sites (listed in Table 1). The active site is accessed by the first substrate, HS-CoM, through a narrow conduit that becomes sterically occluded upon the subsequent binding of CoB-SH. At the base of this channel, the non-covalently bound F_430_ cofactor is positioned to coordinate the substrate. Specifically, the sulfonate moiety (SO_3_^−^) of HS-CoM coordinates with the F_430_ center and is further stabilized by a robust electrostatic network: it forms a salt bridge with the guanidinium group of Mcrγ Arg120 and a H-bond to the peptide nitrogen of Mcrα Tyr444. This anchoring mechanism ensures the precise orientation of the substrate within the catalytic pocket. The conformational flexibility of the F_430_ cofactor is strictly constrained to minor out of plane displacements by an extensive H-bonding and van der Waals network with the *M. ruminantium* Mcr pocket. This structural rigidity is maintained by a multi subunit coordinate environment (Figure 2 and Table 1), involving Mcrα subunits of Val327, Gln331, and Tyr332, alongside Mcrβ subunits of Ile366, Tyr367. Furthermore, the positioning is stabilized by Mcrγ subunits of Ser118, Gly119, Val155 and His156, with additional distal support provided by Mcrα′ subunits of Ala145, Val146, Gln147, and Gln230 residues from the adjacent heterotrimer. These interactions collectively anchor the pyrrole ring plane, ensuring the precise geometric orientation required for catalysis. The F_430_ cofactor is anchored by an extensive H-bonding network, in which fifteen distinct H-bonds are established between its peripheral carboxylate moieties and the peptide amide nitrogens of the Mcr protein backbone.

**Figure 3:**
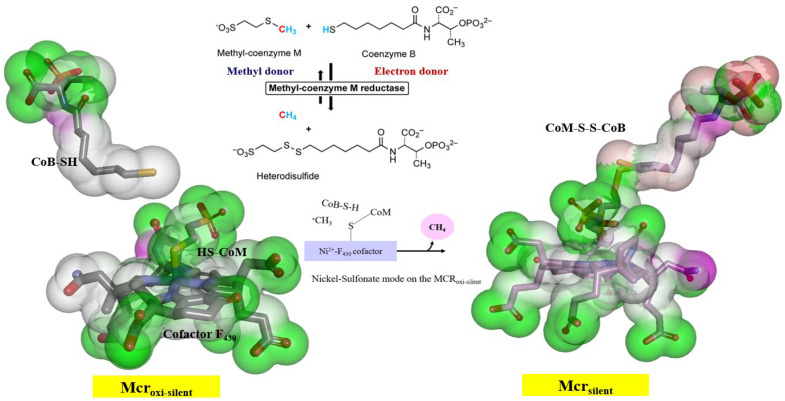
Comparative H-bond surfaces and substrate ligation across two redox states of the *M. ruminantium* Mcr active site. The Mcr_oxi-silent_ state represents the enzyme upon binding to the co-substrates HS-CoM and CoB-SH [26,39]. In the Mcr_silent_ state, the catalytic pocket accommodates the CoM-S-S-CoB heterodisulfide, with concomitant conformational rearrangements observed in the vicinity of the F_430_ cofactor. The axial coordination site of the Ni center, situated above the F_430_ tetrapyrrole plane, is occupied by the thiol group of HS-CoM in the Mcr_oxi-silent_ complex and by a sulfonate oxygen of the hetrodisulfide product in the Mcr_silent_ structure. Structural superposition reveals that the CoB-SH moiety remains precisely aligned between states; however, a subtle reorientation of the sulfur of CoB-SH toward HS-CoM facilitates heterodisulfide coupling, inducing a directional shift in HS-CoM toward CoB-SH in the Mcr_silent_ State.

Specifically, His156 of the Mcrγ subunit may facilitate charge compensation relative to the redox state of the nickel-F_430_ cofactor. In this configuration, the nickel ion positioned distal to the tetrapyrrole ring plane is coordinated by the thiol group of HS-CoM in the Mcr_oxi-silent_ state, and by a sulfonate oxygen of the CoM-S-S-CoB heterodisulfide in the Mcr_silent_ state. These observations are consistent with previously reported findings [40], as illustrated in Figure 3. The active site geometry restricts the rotation of HS-CoM within the narrow segment of the channel. In this orientation, its thiol group (-SH) interacts with the nickel-F_430_ cofactor via H-bonding to the phenolate oxygen atoms of Mcrα Tyr332 and Mcrβ Tyr367 form *M. ruminantium* summarized in Table 1. In contrast, the second substrates, CoB-SH binds within the narrowest segment of the channel formed by the Mcr α, α′, β and α′, α, β′ subunits. This binding effectively shields the active site, preventing the entry of additional substrates. Coenzyme B is anchored via salt bridges between its negatively charged threonine phosphate moiety and a cluster of five positively charged residues, Mcrα Arg270, Mcrα′ subunit of Arg225, Lys256, His257, and Mcrβ His379 residues (in Table 1). The threonine phosphate group of CoB-SH is coordinated by the cationic Mcrα Arg270 residue. Meanwhile, the amide nitrogen of Mcrα Asn481 is oriented toward the sulfur atom of CoB-SH, facilitating H-bonding. Specifically, the donor–acceptor geometry allows the sulfur of CoB-SH to accept H-bonds from amide and peptide nitrogens of Mcrα Asn481 and Val482, respectively (Table 1). These H-bonds likely play a critical role in catalysis by promoting the deprotonation of CoB-SH. This stabilizes the formation of the thiolate anion (CoB-S^−^), which is otherwise energetically unfavorable in solution (pKa > 10 [34]). Furthermore, the heptanoyl arm of CoB-SH is stabilized via van der Waals interactions with a hydrophobic pocket comprising Mcrα (Leu319, Met323, Phe329, Phe443, Met480) and Mcrβ (Phe361, Phe362, and Tyr367) as listed in Table 1.

The optimal complex poses of Mcr_oxi-silent_ with all ligands were employed to investigate the intermolecular interactions and the ensemble of steric and electrostatic features within the Ni-F_430_, HS-CoM, and CoB-SH binding sites. These structural configurations are essential for triggering the catalytic formation of methane. The reduction of CH_3_-S-CoM (K_m_ = 0.7 ± 0.2 mM) and CoB-SH (K_m_ = 0.2 ± 0.1 mM) into CH_4_ and CoM-S-S-CoB occurs with a specific activity of up to 100 µmol min^−1^ mg^−1^ protein (V_max_), consistent with reported values [38]. In the context of Mcr–substrate interactions, the Michaelis constant K_m_ values serve as indicators of enzymatic affinity. The smaller K_m_ for CoB-SH relative to CH_3_-S-CoM demonstrates that CoB-SH possesses a superior affinity for the Mcr enzyme. Consequently, while CoB-SH achieves half-maximal velocity at lower concentrations, CH_3_-S-CoM requires higher concentrations to saturate the enzyme, reflecting its comparatively weaker intermolecular association with the Mcr active site [41].

**Figure 4:**
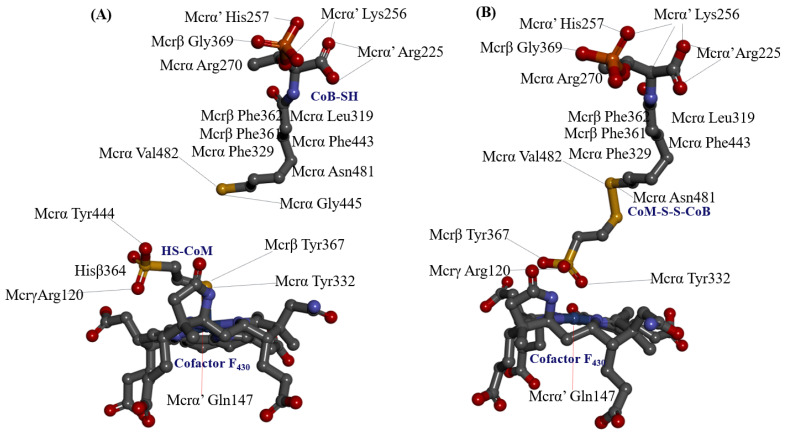
Comparison of Mcr active site interaction in distinct redox states. (**A**) The Mcr_oxi-silent_ state: The coenzyme HS-CoM is bound within the active site that is positioned near the Ni^2+^-F_430_ cofactor, with HS-CoM and CoB-SH spatially separated. (**B**) The Mcr_silent_ state: The catalytic pocket accommodates the CoM-S-S-CoB heterodisulfide product bound to the Ni^2+^-F_430_ cofactor. In the Mcr_silent_ state, the CoB-SH moiety aligns closely with the CoB moiety of the oxidized CoM-S-S-CoB product; the only deviation is a subtle reorientation of the sulfur atom toward HS-CoM to facilitate hetrodisulfide formation in contrast to the CoB-SH conformation, the HS-CoM is rotated 90°, enabling a Ni-sulfonate coordination that is critical for forming the CoM-S-S-CoB heterodisulfide bond.

In the docked poses of Mcr_oxi-silent_ with various ligands, the substrate analog HS-CoM appears to mimic the binding orientation of CH_3_-S-CoM, specifically regarding the sulfonate moiety rather than the thiol group. Consequently, HS-CoM acts as a competitive inhibitor of CH_3_-S-CoM for the Mcr enzyme [28]. The docked configurations of HS-CoM with the Ni-F_430_ cofactor, along with subsequent CoB-SH poses, were evaluated using a structure-based 3D pharmacophore model derived from Mcr_oxi-silent_ complexes, focusing on the distinctive AAADDNNN pharmacophoric features (Table 1). To characterize the pharmacophores illustrated in Figure 4A, the non-bonded interactions within the Mcr_oxi-silent_/all ligands’ complexes were quantified. The analysis revealed a total of 52 favorable interactions, including five electrostatic (charge-based) interactions, four hydrophobic contacts, and 42 hydrogen bonds, while only two unfavorable interactions were identified.

**Figure 5:**
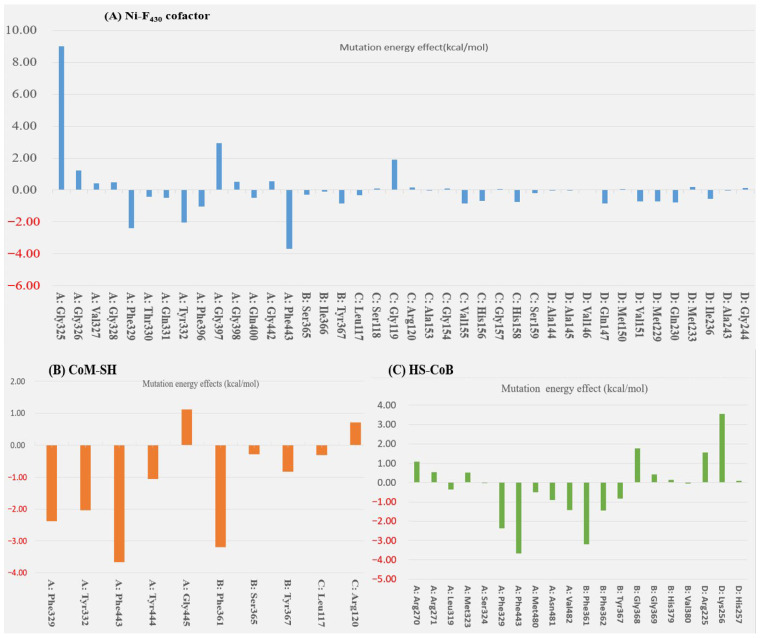
Computational alanine-scanning analysis of the *M. ruminantium* Mcr_oxi-silent_ ternary complex. The impact of single-point mutations on the binding free energy was evaluated for (**A**) the Ni-F_430_ cofactor, (**B**) co-substrate HS-CoM and (**C**) co-substrate CoB-SH. Each key residue within the active site of the ordered ternary complex was systematically substituted with alanine to identify critical binding determinants. The energetic effect of each mutation (**ΔΔ*G_binding_***) is classified into three categories: stabilizing (**ΔΔ*G*** < −0.5 kcal/mol), neutral (−0.5 ≤ **ΔΔ*G*** ≤ 0.5 kcal/mol), and destabilizing (**ΔΔ*G*** > 0.5 kcal/mol). Positive values indicate a loss of binding affinity, highlighting residues essential for substrate and cofactor anchoring.

A total of 21 pairs of directional H-bond donors and acceptors from the Ni-F_430_ cofactor, along with the substrates HS-CoM and CoB-SH, were mapped onto 32 surrounding residues of Mcr_oxi-silent_ within a 3 Å distance. To refine the binding pose predictions, the effects of alanine mutations at each position were utilized to prioritize interaction patterns based on specific chemical features (illustrated in Figure 5 and Tables S3 and S4). The encoded interactions within the Mcr_oxi-silent_ receptor’s bioactive conformation (Figure 4A) were chemically deciphered, identifying 10 critical H-bond partner residues across the subunits: Mcrα (Arg270, Tyr444), Mcrβ (Ser365, Gly369), Mcrγ (Ser118, Gly119, Arg120, Gly154), and Mcrα′ (Arg225, Lys256). These residues represent key interaction points for the 21 identified H-bonds between the two coenzymes and Mcr_oxi-silent_ during the proposed Ni-F_430_ nucleophilic attack. Notably, the binding of the HS-CoM sulfonate moiety is mediated by a H-bond accepted from the main chain nitrogen of Mcrα Tyr444. This specific interaction is strengthened in the Ala mutant compared to the wild-type enzyme, exhibiting a stabilizing effect of −1.06 kcal/mol with the protonated sulfonate group. A salt bridge is formed between the negatively charged sulfonate group of HS-CoM and the guanidinium group of Mcrγ Arg120. This interaction is destabilized by 0.71 kcal/mol upon alanine mutation, primarily due to the loss of positive charge compensation. Furthermore, the phenolate oxygen atoms of Mcrα Tyr332 and Mcrβ Tyr367 within the Mcr_oxi-silent_ complex serve as H-bond acceptors for the thiol group of HS-CoM, which is oriented toward the axial position of Ni-F_430_. The relative importance of these residues is reflected in the differing stabilizing effects observed for the Mcrα Tyr332Ala and Mcrβ Tyr367Ala mutants. In the native of Mcr_oxi-silent_, two tyrosine residues_,_ together with Mcrα Phe329 and Phe443 and Mcrβ Phe361, form an annular arrangement that constitutes the narrowest segment of the substrate channel. Interestingly, the Mcrα Tyr332Ala variant exhibits a significantly greater stabilizing effect (−2.04 kcal/mol) compared to the Mcrβ Tyr367Ala variant (−0.83 kcal/mol). This divergent energetic response suggests that these identical tyrosine residues contribute inequivalently to the channel’s steric environment, likely due to their distinct spatial orientations and the resulting differences in steric widening upon mutation.

Notably, CH_3_-S-CoM is not reduced to CH_4_ by the active Mcr enzyme in the absence of CoB-SH. Consistently, the pentamethyl ester of the F_430_ cofactor exhibits no reactivity toward the CH_3_-S-CoM substrate, as previously reported [42]. The reduction reaction requires the simultaneous presence of CoB-SH alongside either the CH_3_-S-CoM substrate or the active Ni-F_430_ cofactor. Specifically, the negative ionizable groups of the threonine phosphate moiety in CoB-SH are anchored by a cluster of positive charge features near the entrance of the Mcr_oxi-silent_ substrate channel. This stabilization is mediated by a network of salt bridges involving five key basic residues: Mcrα Arg270 and Arg271, Mcrα′ Arg225 and Lys256, and Mcrβ His379. Regarding the interaction preferences of the salt bridges, alanine mutations resulting in chemically inert charge centers are expected to destabilize the binding of the threonine phosphate moiety of CoB-SH compared to the wild-type positive residues. Specifically, the Arg270Ala and Arg271Ala (Mcrα), Arg255Ala and Lys256Ala (Mcrα′) mutants exhibited significant destabilization effect of 1.08, 0.53, 1.56 and 3.54 kcal/mol, respectively (explicated in Figure 5 and Table S4). In contrast, the His379Ala (Mcrβ) mutant showed a minimal destabilization effect of only 0.15 kcal/mol (Table S4).

On the other hand, the hydrophobic features of CoB-SH are governed by spatial constraints within 5.5 Å of the centroids of several non-polar residues in the Mcrα subunit. These interactions involve Leu319, Met323, Phe329, Phe443, and Met480, which facilitate non-polar contacts as detailed in Table 1 and Figure 4A. The elongated heptanoyl arm of CoB-SH must negative a narrow tunnel formed by an annular arrangement of bulky aromatic residues, specifically Phe329 and Phe443 (Mcrα) and Phe361 and Phe362 (Mcrβ). This configuration imposes significant steric hinderance on the co-substrate. The impact of these residues is evidenced by the alanine mutation data, where the replacement of bulky phenylalanine with smaller hydrophobic side chains relieves steric tension and attenuates the ordered aromatic orientations. This results in substantial stabilizing effects at each position, with energy changes ranging from −1.44 to −3.67 kcal/mol, as detailed in Table S4. The effects of alanine replacement for Mcrα Met323 and Met480 were evaluated, as these residues define the start (Met480) and end (Met323) of the tunnel segment through van der Waals contacts. Interestingly, these mutations exhibited symmetric energetic responses: a stabilizing effect (−0.51 kcal/mol) for one and a destabilizing effect (0.51 kcal/mol) for the other. These findings are consistent with the steric requirements for substituting a methionine side chain with the smaller alanine within the tunnel. Furthermore, the CoB-SH thiol group is positioned to accept a H-bond from either the side chain nitrogen of Asn481 or the main chain nitrogen of Val482 in the Mcrα subunit. This interaction is supported by a donor–acceptor geometry within a distance of 3.5 Å, as identified in the Mcr_oxi-silent_ structure (Table 1). Mcrα Asn481 interacts closely with Mcrβ Phe381, which is located within the highly hydrophobic region of the substrate channel between the sulfur atoms of HS-CoM and CoB-SH. Comparing the chemical properties of polar Asn481 and nonpolar Val482, the Asn481Ala mutation exhibits a more pronounced stabilizing effect (−1.42 kcal/mol) than the Val482Ala mutation (−0.91 kcal/mol), both occurring within a hydrophobic structural context. While our computed model for alanine mutation effect is robust, traditional mutagenesis experiments remain challenging for Mcr. This difficulty contributes to a scarcity of enzymological data, primarily due to the enzyme’s high susceptibility to oxidative inactivation and the extreme lability of the active Ni^+^ or Ni^3+^ forms of the F_430_ cofactor.

### 2.2. Decisive Functional Residues for Active-Site Ligand Binding in Mcr_silient_ State

**Figure 6:**
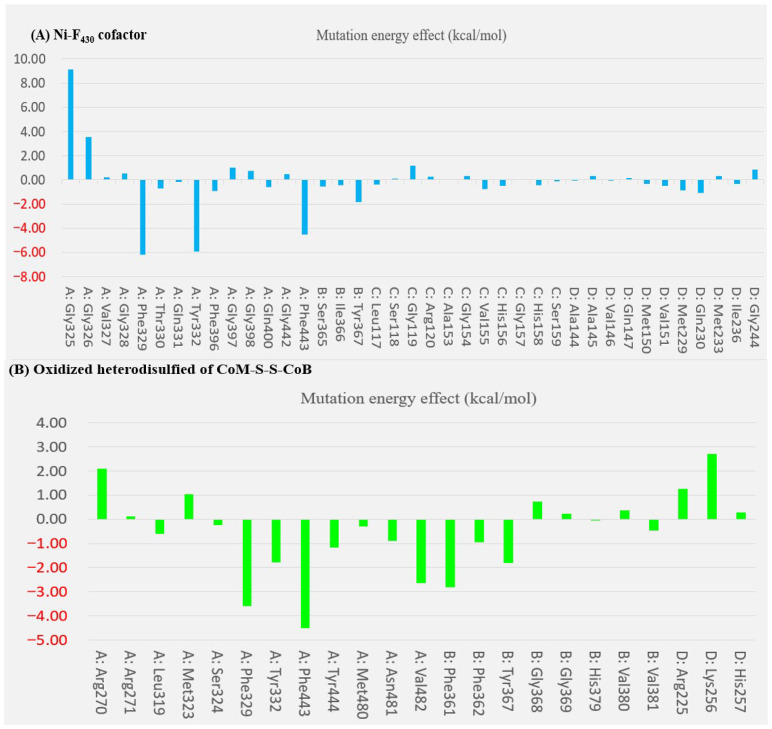
Computational alanine-scanning analysis of the *M. ruminantium* Mcr_silent_ complex. The impact of single-point mutations on binding free energy was evaluated for (**A**) the Ni-F_430_ cofactor and (**B**) the heterodisulfide product CoM-S-S-CoB. Each key residue within the active site of the ligand-bound Mcr_silent_ model was systematically substituted with alanine to identify critical binding determinants. A mutation energy (**ΔΔ*G_binding_***) greater than 0.5 kcal/mol is classified as destabilizing, indicating a loss of interaction strength. Conversely, negative **ΔΔ*G_binding_*** values represent stabilizing effects, where binding affinity increases upon side-chain substitution with alanine.

In the *M. ruminantium* Mcr_silent_ model, the heterodisulfide binding of HS-CoM to CoB-SH triggers an induced fit mechanism around the substrate-binding sites situated above cofactor F_430_, as illustrated in Figure 3 and Figure 4B. Superposition of the template structures (PDB IDs: 1HBN and 1HBM) reveals that reduced coenzyme B (CoB-SH) and the CoB moiety of the heterodisulfide (CoM-S-S-CoB) occupy nearly identical positions within the Mcr_silent_ model; the only notable deviation is a slight rotation of the CoB-SH sulfur atom toward HS-CoM [40]. In contrast to CoB-SH, a 90° rotation of HS-CoM shifts its thiol group perpendicular to the F_430_ cofactor, while the sulfonate group (SO_3_^−^) remains parallel to the F_430_ tetrapyrrole plane. This spatial rearrangement brings the sulfur atoms of both substrates into close proximity, facilitating heterodisulfide formation despite the weakened H-bonding between the sulfonate moiety of HS-CoM and the Mcrα residues Asn481 and Val482 residues (in Figure 4B). The overall architecture of the *M. thermautotrophicus* Mcr templates is highly conserved between the Mcr_oxi-silent_ (1HBN) and Mcr_silent_ (1HBM) crystal structures shown in Figure S10 and Table S2. Structural discrepancies are primarily confined to the redox states of the CoB-SH and HS-CoM substrates. Notably, both template structures represent inactive Mcr states, characterized by a Ni^3+^- F_430_ cofactor oxidation state. Upon the transition to the *M. ruminantium* Mcr_silent_ model, the movement of HS-CoM toward CoB-SH positions the sulfur atom of the resulting CoM-S-S-CoB product within a pocket flanked by nonpolar aromatic residues, including Mcrα Phe329, Tyr332, Phe443 and Mcrβ Phe361, Tyr367. These hydrophobic residues from a conserved annular arrangement observed in both the Mcr_oxi-silent_ and Mcr_silent_ structures, as well as across all other template Mcr sequences. In the active site, the sulfur atom of CoB-SH loses its anchoring H-bond with Mcrα Asn481, shifting its primary interaction toward Mcrα Val482 (Figure 4B). Consequently, the sulfonate moiety of HS-CoM coordinates with Mcrα Phe443, while the sulfonate moiety shifts its H-bonding engagement to Mcrα Tyr332 (represented in Table 1 and Table 2). The F_430_ cofactor remains rigidly anchored within the Mcr models across different oxidation states, both before and after the reduction of HS-CoM with CoB-SH. In the Mcr_silent_ model, the five carboxylate groups of F_430_ are stabilized by an extensive H-bonding network involving Mcrα (Ala145, Val146, Gln331, Tyr332) and Mcrα′ (Ser118, Gly119, Gly154, Val155, His156, His158) residues. These interactions effectively neutralize the anionic charge of the carboxylate groups through localized electrostatic stabilization (in Figure 2). The optimal positioning of the CoB-SH sulfhydryl group does not imply a direct coordination with the proximal nickel center, but rather ensures its immediate proximity to the methyl group of CH_3_-S-CoM (Figure 3 and Figure 4B). Consequently, methane formation from these substrates is facilitated by the hydrophobic aromatic microenvironment, which acts as a driving force to expel water molecules from the catalytic channel pocket. The annular rearrangement of hydrophobic aromatic residues, combined with the increased flexibility of the glycine-rich stretch (Mcrβ 366–372) within the CoB-SH channel, enables CoB-SH to penetrate deeper into the substrate channel upon CH_3_-S-CoM binding. This structural transition effectively positions the thiol group of CoB-SH in closer proximity to the Ni^3+^ center within the active site. The role of HS-CoM induced conformational changes as a driving force for substrate reduction is elucidated by comparing the effects of alanine mutations before and after disulfide formation. This mechanism is further supposed by a comprehensive analysis of ligand complexes in both the Mcr_oxi-silent_ and Mcr_silent_ states (in Figure 4B and Figure 6). A defining distinction between these two Mcr states lies in the molecular gating mechanism, which governs substrate specificity based on their physicochemical properties. During the reductive process, specifically the activation of CH_3_-S-CoM, the reactive HS-CoM undergoes a displacement of over 4 Å from its initial position in the Mcr_oxi-silent_ complex toward CoB-SH. This movement brings the thiol sulfur atoms of both coenzymes into van der Waals contact prior to the formation of the covalent CoM-S-S-CoB heterodisulfide. The catalytic center, featuring a Ni^3+^ ion situated on the distal side of the F_430_ tetrapyrrole plane, is flanked by a cluster of hydrophobic and aromatic residues (e.g., Mcrα Phe329, Phe443, Val482, Tyr332 and Mcrβ Phe361, Tyr367). These residues define the substrate channel between the Ni ion and the thiol group of CoB-SH, effectively encapsulating the sulfur atoms of the resulting heterodisulfide.

In the transition from the Mcr_oxi-silent_ to the Mcr_silent_ state, the HS-CoM sulfonate moiety dissociates from Mcrγ Arg120, which is otherwise anchored to the F_430_ lactam nitrogen (Table 2 and Table S5). Structural comparison reveals that this interaction is abrogated as Mcrα Tyr332 and Mcrβ Tyr367 undergo shifts to occupy new positions within the Mcr_silent_ ligand-bound model (in Figure 4). Concomitant with a 90° rotation of HS-CoM, the sulfonate group of the CoM-S-S-CoB hetrodisulfide undergoes a H-bonding shift: one oxygen atom bonds to the hydroxyl group of Mcrα Tyr332, while another interacts with Mcrβ Tyr367, replacing the H-bond donor interaction with the thiol group of HS-CoM observed in the Mcr_oxi-silent_ state. The phenolic oxygen of Mcrβ Tyr367, which accepts a H-bond from the F_430_ lactam nitrogen, is predicted to be more acidic than Mcrα Tyr332 due to its specific orientation and local conformational environment, as outlined in Table 2 and Figure 4B. Mcr achieves long-range electron transfer (10–15 Å) from the Ni^+^-F_430_ center to the coenzyme substrates through a tyrosine-mediated relay [43]. This process is coupled with an induced-fit mechanism involving the Mcrβ Gly368-Gly369 loop, which reorients upon CoB-SH binding. In the Mcr_silent_ complex, the sulfonate moiety of HS-CoM characterized by a higher activity (pKa 1.9) than CH_3_-S-CoM (pKa 2.5) due to diminished inductive electron donation interacts with the conserved Tyr332 and Tyr367 residues and both Gly368 and Gly369 residues. This electrostatic environment, shaped by the strategic positioning of coenzymes, significantly boosts the catalytic efficiency shown in Figure 4. Substrate-induced fit in Mcr is mediated by a flexible Gly-rich loop, analogous to those found in ATP-binding proteins [44]. This transition triggers structural rearrangement of Mcrα Asn481 and Val482, specifically flanking the CoB-SH thiol position. While Asn481 promotes thiol deprotonation for hydrogen transfer, its interaction with the sulfur moiety is abolished upon heterodisulfide formation, whereas Val482 remains anchored. The coupling of the thiol deprotonation of CoB-SH notwithstanding its unfavorable (pKa of 9.2 vs. 9.4 for Hs-CoM [43]) with the exergonic and irreversible production of methane drives the reaction forward in the Mcr_silent_ complex.

The contribution of key aromatic and glycine residues to the catalytic driving force was quantified via alanine scanning on Mcrα Tyr332, Mcrβ Tyr367, and the Gly368 and Gly369 within the Mcrβ subunit. Structural analysis revealed that the H-bonding stereochemistry of Tyr332 and Tyr367 is suboptimal for proton acceptance, despite Tyr332’s spatial advantage near the Ni^3+^ center. The lack of H-bond engagement for the Tyr332 phenolic oxygen correlates with its reduced acidity. Mutation of Tyr367 to Ala resulted in a significant stabilization gain of −1.81 kcal/mol in the Mcr_silent_ model, a substantial increase from the −0.83 kcal/mol observed in the Mcr_oxi-silent_ state. In contrast, the stabilizing influence of the Tyr332Ala mutation decreased from −2.04 kcal/mol in the Mcr_oxi-silent_ from to −1.79 kcal/mol in the Mcr_silent_ form, highlighting its distinct role in state-specific stabilization. The acidity differential between Mcrα Tyr332 and Mcrβ Tyr367 is reflected in the subtle ΔΔG variation (−0.02 kcal/mol) observed upon alanine substitution. The Gly-rich loop (Mcrβ 366–372) undergoes essential conformational reorientation, specifically a peptide bond flip across the Tyr367-Gly368-Gly369 segment, to accommodate the Mcr_silent_ transition. While Gly368 anchors the loop’s start and Gly368 stabilizes the CoB-SH moiety via H-bonding, the Tyr367 remains engaged with HS-CoM component of the heterodisulfide. Alanine scanning reveals that Gly368 is vital for loop stability, its mutant to alanine introduces significant steric hindrance and an entropic penalty (0.73 kcal/mol). Conversely, the Gly369Ala variant remains energetically neutral (0.24 kcal/mol), underscoring the site-specific sensitivity of the Gly-rich loop to side-chain perturbations (Table S6). Alanine scanning reveals that the destabilization incurred by the Mcrβ Gly368Ala mutation decreases from 1.78 kcal/mol in the Mcr_oxi-silent_ complex to 0.73 kcal/mol in the Mcr_silent_ form. This 2.4-fold decrease in energetic penalty suggests a state-specific role for Mcrβ Gly368. We hypothesize that this effect is linked to the reorientation of Mcrβ Tyr367, which dissociates from the F_430_ pyrrole nitrogen and the axial Ni center in response to the alternative conformational state of the Gly-rich loop. H-bonding between Mcrβ Tyr367 and cofactor F_430_ is a critical determinant of the structural transition within the Mcr_silent_ active site. Alanine-scanning mutagenesis reveals that the stabilization derived from electrostatic interactions and hydrophobic effects is substantially amplified in the Mcr_silent_ form (−1.81 kcal/mol) relative to the Mcr_oxi-silent_ form (−0.83 kcal/mol). This augmented stability in the Mcr_silent_ state is attributed to the preservation of the essential H-bond network centered around the Ni-F_430_ cofactor (Tables S3 and S5). The Mcrβ Gly368Ala mutation shows a consistently neutral energetic profile, transitioning from 0.42 kcal/mol in the Mcr_oxi-silent_ state of 0.24 kcal/mol in the Mcr_silent_ state (Tables S4 and S6).

Structural analysis of the Mcr_silent_ state reveals that the Gly rich loop (Gly368-Gly369) fails to establish strong H-bonds with the HS-CoM moiety, especially when compared to the robust interactions of Mcrα Asn481 and Val482 with the CoB-SH moiety. This attenuated bonding is primarily due to the disordered donor–acceptor alignment inherent in the loop’s local conformation. Alanine substitution at the Mcrα Val482, which reduces the side-chain volume (Isopropyl to methyl), yields a high stabilization energy (ΔΔG = −2.64 kcal/mol) due to optimized H-bonding geometry. Conversely, the Asn481Ala mutation, which neutralizes a local positive charge with a nonpolar group, attenuates stabilization to only −0.88 kcal/mol. This disparity underscores the distinct functional roles of Val482 (steric fit) and Asn481 (electrostatic interaction) in the active site, as outlined in Figure 6 and Table S6. Assessment of acid dissociation constants at pH7.4 demonstrates that the Asn481Ala mutation induces a marked shift in pKa values (from −28 to 41), reflecting a stronger acidic profile than that of the Val482Ala variant (which shifted from 76 to 41) [45]. This transition to opposing chemical properties suggests that Asn481 is a key determinant in stabilizing the proton-donor state within the Mcr active site.

**Figure 7:**
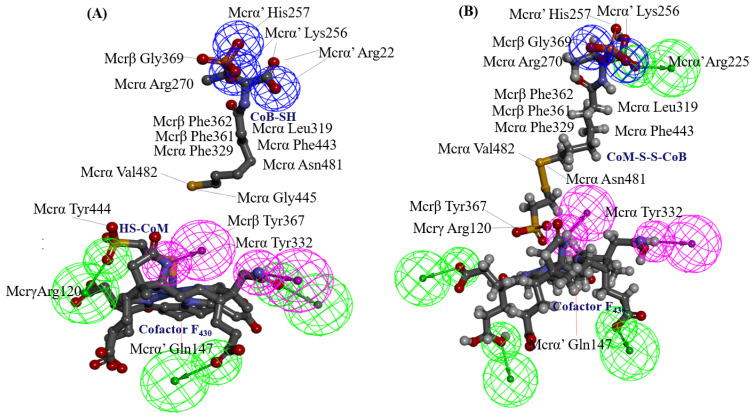
Structure-based pharmacophore models of *M. ruminantium* Mcr-coenzyme complexes. Pharmaocphore models were derived for two distinct inactive catalytic states: (**A**) the Mcr_oxi-silent_ state (oxidized, co-substrate model) and (**B**) the Mcr_silent_ state (reduced, product-bound) (**A**) an eight-feature model (AAADDNNN) for the HS-CoM/CoB-SH bound state illustrates, comprising three H-bond acceptors (HBA, green spheres), two H-bond donors (HBD, purple spheres), and three negative ionizable centers (blue spheres). (**B**) the eight-features model (AAAADDNN) for heterodisulfide-bound state displays, consisting of four H-bond acceptors, two H-bond donors, and two negative ionizable centers. These discrete spatial arrangements highlight the conformational and electrostatic shifts occurring within the active site during the catalytic cycle.

To circumvent the lack of structural information on the *M. ruminantium* Mcr receptor, 3D pharmacophores were derived from the bioactive conformers of individual coenzymes. Given that coenzymes exhibit state-specific binding orientations depending on their oxidation levels during CH_4_ production, we developed discrete pharmacophore models to account for these non-overlapping binding modes. The chemical properties and spatial arrangements of the coenzyme analogues were systematically extracted based on the two H-bonds corresponding to the sulfonate and thiol groups of HS-CoM in Figure 4A. Interaction between the thiol of HS-CoM and the Ni^+^-F_430_ cofactor occurs exclusively in the presence of CoB-SH. Furthermore, the Mcr reduction model was refined for each pharmacophore feature, specifically adjusting the seven-carbon mercaptoalkanoyl chain to adopt an elongated, hydrophobic conformation. As presented in Figure 4, the threonine phosphate moiety exerts significant electrostatic influence through its three ionizable centers, facilitating interactions within the hydrophobic substrate channel. Concurrently, the thiol group of CoB-SH is strategically oriented toward the Ni atom, satisfying the requisite H-bonding geometry for the catalytic complex. To elucidate the mechanism of heterodisulfide (CoM-S-S-CoB) formation, we defined a comprehensive pharmacophore (AAAADDNN) within the *M. ruminantium* Mcr_silent_ binding pocket. The model incorporates a network of 108 total interactions, specifically categorized into 49 favorable, 12 unfavorable, 3 electrostatic, 9 hydrophobic, and 35 H-bond features. These parameters facilitate a detailed structural interpretation of the catalytic transition from HS-CoM and CoB-SH substrates (illustrated in Figure 4B). A total of 62 interactions were initially captured by the structural probes (in Table 2). To identify the key driving forces for CoM-S-S-CoB binding within the Ni-F_430_ environment, these features were pruned to focus on the most energetically favorable contacts. The resulting interaction map was delineated based on the energetic shifts observed in alanine mutation studies, as detailed in Figure 6 and Tables S5 and S6. The primary substrate-specific affinities were localized to nine key binding points, including Mcrα (Arg270, Phe329, Tyr332, Met480), Mcrβ (Phe361, Tyr367), Mcrγ (Gly112, Gly154) and Mcrα′ (Arg225) subunits. These residues are crucial for substrate recognition and define the descriptive binding mode of CoM-S-S-CoB. To facilitate virtual screening of anti-methanogenic candidates, we refined the interaction features into an optimal assemblage of eight pharmacophore features: AAADDNNN for the Mcr_oxi-silent_ complex (Figure 7A) and AAAADDNN for the Mcr_silent_ complex (Figure 7B). A sufficient number of features ensures selectivity, while excessive features become overly restrictive and increase the likelihood of false negatives [46,47]. CoM-S-S-CoB acts as a feedback inhibitor for the reductive activation of Mcr, a process regulated by the heterodisulfide reductase system. This system, which employs iron–sulfur proteins for cytoplasmic bifurcation or cytochrome-mediated electron transfer [48], ensures the recycling of CoM-SH and CoB-SH from the heterodisulfide product. Leveraging the detailed physicochemical properties of the Mcr ligand-bound structures, we established a framework for pharmacophore modeling to discover novel anti-methanogenic agents with high efficacy.

## 3. Discussion

The structural elucidation of the *M. ruminantium* Mcr complex reveals a sophisticated gating mechanism dictated by hydrophobic and electrostatic constraints. Our model of Mcr_oxi-silent_/all ligand complexes provides a structural rationale for the long-observed requirement of both coenzymes Hs-CoM and CoB-SH for Mcr activity. The reduction of CH_3_-S-CoM to methane by the Mcr enzyme is strictly dependent on the presence of CoB-SH, as neither CH_3_-S-CoM nor the pentamethyl ester of the F_430_ cofactor reacts in isolation. To understand this cooperatively, the binding of the threonine phosphate moiety on the CoB-SH at the substrate channel entrance was characterized. This moiety is anchored by a positive charge cluster comprising five basic residues: Mcrα Arg270, Arg271; Mcrα′ Arg225, Lys 256; and Mcrβ His379. In silico alanine scanning revealed that neutralizing these charges significantly destabilizes binding. The Mcrα′ Lys256Ala mutation exerted the most profound destabilizing effect (3.54 kcal/mol), followed by Mcrα′ Arg225Ala (1.56 kcal/mol) and Mcrα Arg270Ala (1.08 kcal/mol). Conversely, the Mcrβ His379Ala mutation resulted in a negligible energetic shift (0.15 kcal/mol), suggesting a subordinate role in phosphate stabilization. The heptanoyl arm of CoB-SH occupies a hydrophobic tunnel formed by an aromatic ring of residues, including Mcrα Phe329, Phe443 and Mcrβ Phe361, Phe362. Alanine substitutions of these phenylalanine residues resulted in marked stabilizing effects, ranging from −1.44 to −3.67 kcal/mol, suggesting that the native aromatic side chains impose significant steric constraints to ensure precise substrate orientation. Within this segment, Mcrα Met323 and Met480 flank the tunnel; their mutations exhibited mirrored energetic profiles (stabilizing −0.51 kcal/mol vs. destabilizing 0.51 kcal/mol), consistent with the steric demands of the methionine to alanine transition. At the catalytic core, the thiol group of CoB-SH is positioned to accept H-bonds from the Mcrα Asn481 side chain and the Val482 main chain. Notably, the Asn481Ala mutation showed a greater stabilizing effect (−1.42 kcal/mol) compared to Val482Ala (−0.91 kcal/mol), despite both participating in the hydrophobic structural context near the sulfur atoms of HS-CoM and CoB-SH.

The intricate salt-bridge network at the channel mouth act as a selective filter, where the destabilization energies observed in the Mcrα′ Lys256 and Arg225 mutants highlight these residues as critical gatekeepers for CoB-SH entry. The disproportionate energy shifts among these basic residues suggest that the Mcrα′ subunit plays a dominant role in anchoring the second substrate, effectively locking the active site catalysis. The “steric hinderance” identified in the hydrophobic tunnel evidenced by the increased stability of alanine variants suggests a highly evolved mechanism for substrate alignment. The aromatic residues (Mcrα Phe329, Phe443, and Mcrβ Phe361, Phe362) likely serve as a molecular “corset” that constraints the flexible heptanoyl arm of CoB-SH into a bioactive conformation, prioritizing catalytic precision over binding affinity. Furthermore, the stabilizing observed in the Asn481Ala mutant suggests that while Asn481 is essential for H-bond donation to the thiol of CoB-SH, its native polar side chain exists in a precarious balance within the highly hydrophobic environment near Mcrβ phe381. This effectively partitions the substrate channel between the HS-CoM and CoB-SH sulfur atoms, creating a fine C-S bond cleavage.

The catalytic efficiency of Mcr in *M. ruminantium* relies on a highly coordinated “driving force” generated by hydrophobic aromatic arrangements and conformational flexibility. The transition of Mcr_oxi-silent_ to the Mcr_silent_ state is characterized by a significant induced-fit mechanism. Superposition of *M. ruminantium* model with template structures (PDB 1HBN and 1HBM) reveals that while the CoB-SH moiety remains relatively stationary, HS-CoM undergoes a dramatic 90° rotation. This rotation shifts the thiol group of HS-CoM perpendicular to the F_430_ cofactor and moves the molecule over 4 Å toward CoB-SH, facilitating van der Waals contact between the sulfur atoms prior to heterodisulfide CoM-S-S-CoB formation. The binding of HS-CoM is stabilized by a redefined H-bonding network. In the Mcr_silent_ state, the sulfonate moiety of HS-CoM loses its interaction with Mcrγ Arg120 and instead coordinates with Mcrα Tyr332 and Mcrβ Tyr367. Notably, the hydroxyl group of Mcrβ Tyr367 is H-bonded to the lactam nitrogen of F_430_, enhancing its acidity and facilitating long-range (10–15 Å) H^+^-coupled electron transfer from the Ni center to the thiolate of HS-CoM. The results demonstrate that the 90° rotation of HS-CoM is not merely a spatial adjustment but a critical gatekeeping mechanism that aligns the substrate for heterodisulfide (CoM-S-S-CoB) formation.

The CoB-SH binding site is regulated by a flexible glycine-rich loop (Mcrβ 366–372). The role of the Mcrβ 366–372 of glycine-rich loop mirrors induced-fit mechanisms seen in ATP-binding proteins, suggesting a conserved evolutionary strategy for managing flexible substrate channels. A peptide bond flip involving Mcrβ Gly368 and Gly369 is essential for catalytic readiness. The energetic cost of Gly368Ala mutation suggests that loop flexibility is finely tuned to allow CoB-SH to penetrate deep enough into the channel to interact with the Ni-F_430_ center. Ala-scanning mutagenesis highlights the critical role of these residues: Mcrβ Gly368Ala results in a significant destabilizing effect (0.73 kcal/mol), likely due to steric hindrance and entropic penalties within the loop. Mcrα Val482Ala exerts a stabilizing effect of −2.64 kcal/mol by optimizing H-bonding distances. Mcrα Asn481Ala abolishes a vital H-donor interaction, resulting in a significantly lower stabilizing effect (−0.88 kcal/mol) and a dramatic shift in predicted pKa (from −28 to 41) underscoring its role in facilitating the deprotonation of CoB-SH. The increased acidity of Mcrβ Tyr367, induced by its interaction with the lactam nitrogen of F_430_, identifies it as a primary conduit for the redox reaction. The coupling of the unfavorable deprotonation of CoB-SH (pKa 9.2) with the irreversible formation of methane ensures the reaction proceeds forward under physiological conditions.

We generated coenzyme-based 3D pharmacophores to define the bioactive conformers. The Mcr_silent_ state/all ligand complexes are best represented by an AAAADDNN feature model (four H-bond acceptors, two H-bond donors, two negative ionizable centers), encompassing 62 unique interactions. Nine key residues (Mcrα: Arg270, Phe329, Tyr332, Met480; Mcrβ Phe361, Tyr367; Mcrγ: Gly112, Gly154; Mcrα′: Arg225) were identified as high-affinity anchor points for substrate recognition. Finally, the transition from an AAADDNNN pharmacophore (Mcr_oxi-silent_) to an AAAADDNN model (Mcr_silent_) provides a refined template for virtual screening (in Figure 7). By targeting the nine key residues anchor points (particularly the hydrophobic pocket formed by the aromatic annulus) future new anti-methanogenic compounds can identify that mimic the inhibitory properties of CoM-S-S-CoB potentially disrupting the hetrodisulfide reductase system and reducing methane emissions in ruminants.

Beyond catalysis, the inhibitory nature of the product CoM-S-S-CoB represents a significant regulatory checkpoint. Science all methanogens rely on a heterodisulfide reductase (Hdr) system [49] for the regeneration of coenzyme via electron bifurcation or cytochrome-dependent pathways, the persistence of CoM-S-S-CoB can potentially suppress the reductive activation of Mcr. This feedback inhibition, combined with our pharmacophore models, provides a robust “chemogenetic map” for the rational design of anti-methanogenic compounds.

Despite these computational insights, traditional mutagenesis of Mcr remains a formidable challenge. A persistent challenge in Mcr enzymology has been the extreme susceptibility of the enzyme to oxidative inactivation. The active Ni(I) state of the F_430_ cofactor is remarkably labile, and traditional site-directed mutagenesis has been largely impeded by the difficulty of maintaining enzymatic activity during purification and characterization. However, our computational framework addresses this critical knowledge gap, providing a high resolution “chemogenetic map” where experimental data are currently scarce. The structure-based pharmacophore and energetic profiles presented here offer a predictive framework for understanding Mcr enzymology, bypassing the current limitations of in vitro site-directed mutagenesis and providing a blueprint for future anaerobic biochemical assays.

To validate the structural and energetic predictions presented in this study, several specialized experimental approaches are warranted. Anaerobic site-directed mutagenesis targeted at the identified high-affinity anchor points (e.g., Mcrα′ Lys256, Mcrβ Tyr367), coupled with isothermal titration calorimetry (ITC) or surface plasmon resonance (SPR), would provide direct measurement of the predicted binding kinetics and destabilization energies. Furthermore, high-resolution cryo-electron microscopy (cryo-EM) could potentially capture the transient induced-fit conformations and 90° rotation of HS-CoM during the transition from the Mcr_oxi-silent_ to the Mcr_silent_ state. To resolve the electronic environment of the Ni-F_430_ center and the long-range H^+^-coupled electron transfer, electron paramagnetic resonance (EPR) spectroscopy remains the gold standard. These integrated biochemical and biophysical assays will be essential to confirm our ‘chemogenetic map’ and facilitate the development of next-generation anti-methanogenic compounds.

Targeting the specific interaction features identified in *M. ruminantium* offers a pathway to develop broad-spectrum inhibitors that are highly effective at low concentrations. By exploiting the volume restrictions and H-bonding patterns unique to the Mcr active site, future virtual screening efforts can identify candidates that prevent methanogen recolonization without compromising the broader rumen fermentation ecosystem. This study thus provides the functional physicochemical information necessary to bridge the gap between structural biology and sustainable methane mitigation strategies in global livestock production.

## 4. Materials and Methods

All molecular modeling and optimization were performed in Discovery Studio, version 2017 R2, from BIOVIA (San Diego, CA, USA) [50].

### 4.1. A Structure Model of Methyl-Coenzme M Reductase from Methanobrevibacter ruminantium

The primary sequence of methyl-coenzyme M reductase (Mcr) from *Methanobrevibacter ruminantium* was retrieved from NCBI database. Phylogenic analysis and sequence alignment via CLUSTALW (Clustal 1.8) confirmed that *M. ruminantium* Mcr belongs to cluster I [24,29], sharing high sequence identify with *Methanothermobacter marburgensis* and *Methanothermobacter thermautotrophicus* (up to 80.4% for the McrA subunit) represented in Figures S6–S8. Given the high conservation (>99% similarity in McrABG subunits), homology models of the *M. ruminantium* Mcr heterohexamer [(*αβγ*)_2_] were constructed. Five high-resolution (1.1–1.9 Å) X-ray crystal structures from cluster I methanogens were selected as templates (Table S1) to represnted two distinct inactive states: the Mcr_oxi-silent_ state (bound to Ni(I)-F_430_, HS-CoM, and CoB-SH) and the Mcr_silent_ state (bound to Ni(III)-F_430_ and CoM-S-S-CoB). The resulting C*_α_* trace RMSD of 0.32–0.52 Å against the templates validated the structural integrity of the models. All modeling and structural superpositions were performed using the iFATCAT rigid-body algorithm [51] to ensure the conservation of the substrate channel and active site architecture. The heterohexameric strucrutual model of *M. ruminantium* Mcr [*α*_2_*β*_2_*γ*_2_] was constructed using MODELER protocol within the Discovery Studio interface (Dassalut Systemes). Initial monomeric units (*α*, *β*, *γ*) were generated and refined through protein threading, utilizing the high-resolution templates from *M. marburgensis* (PDB: 5A0Y) and *M. thermautotrophicus* (PDB: 1HBN) as shown in Figures S9 and S10. The hexameric assembly was organized by applying P2-crystallographic symmetry derived from the templates. Local conformers, particularly loops with high aggregation propensities, were optimized using the CHARMm force field and minimized via the Particle Mesh Ewald (PME) method [52] to eliminate steric clashes. The final model reached a lowest CHARMm potential energy of −388.578 kcal/mol. Structural integrity was rigorously assessed using the Discrete Optimized Protein Energy (DOPE) score [53] and Probability Density Function (PDF) energy [54], ensuring the model satisfied all homology-derived genometric restraints. The refined heterohexamer (2476 residues) exhibited high steric and electrostatic similarity (0.839–0.908) to the templates (Figure S9). Crucially, the 33 Å substrate channel and active site funnels within the Mcr*α* subunit [25,55] remained structurally intact, preserving the functional architecture required for methane transport and substrate binding (Figure S10). The sterochemical quality and internal stability of the *M. ruminantium* Mcr model were evaluated via Ramanchandran plot analaysis (Figure S11). The query model exhibited 96.7% of residues in the favored regions, demonstrating high reliability comparable to the experimental templates (97.0%) as depicted in Figure S11. Minor structural deviations from the templates were primarily localized to the protein surface, particularly regarding the coordination of exogenous ions (Mg^2+^, Na^+^, Zn^2+^) and solvent molecules. While crystallization conditions for templates such as *M. thermautotrophicus* favor salt-mediated stabilization of surface-exposed acidic clusters, our model reflects a native-like mesophilic state with increased surface flexibility. This inherent plasticity in the optimized Mcr model likely facilitates the conformational adjustments required for substrate processing in the rumen environment. The final heterohexameric assembly (Figure 1) provides a robust framework for characterizing the methanogenesis machinery and serves as a starting point for identifying chemogenetic targets.

### 4.2. Complex Models of the Query Mcr Receptor with F_430_ and Substrates in Two Different Enzyme States

To elucidate the substrate recognition mechanism of the *M. ruminantium* Mcr receptor, we performed hierarchical molecular docking of the Ni-F_430_ cofactor, HS-CoM, and CoB-SH coenzymes. Initial ligand poses were generated by superimposing the *M. ruminantium* model onto five high-resolution cluster I templates (Table S2), preserving the conserved substrate funneling architecture. Sequential docking was executed using the CDOCKER algorithm [56,57] under the CHARMm force field, employing a simulated annealing protocol (heating to 700 K, followed by cooling to 300 K) to refine the substrate-induced fit within the active site. The thermodynamic stability of the resulting complexes was evaluated through binding free energy (Δ*G*_bind_) calculations, accounting for translational and rotational entropy via statistical mechanics. The prioritized docking poses yielded favorable CDOCKER scores, with HS-CoM and CoB-SH exhibiting interaction energies −12.34 and −98.94 kcal/mol, respectively (Figure 2 and Figure 3). Notably the binding energy for CoB-SH (Δ*G*_bind_ = −804.56 kcal/mol) was significantly higher than that of HS-CoM, confirming a strictly ordered binding mechanism where HS-CoM must occupy the Ni-proximal site prior to the entry of the second substrate (summarized in Table 3). These results suggest that the rate-limiting step in *M. ruminantium* methanogenesis is governed either by the initial conformational shift in HS-CoM or its subsequent interaction with the Ni center.

To elucidate the conformational dynamics during the final stages of methanogenesis, we performed comparative docking of the heterodisulfied product (CoM-S-S-CoB) into the *M. ruminantium* Mcr receptor. The structural transition from the Mcr_oxi-silent_ to the Mcr_silent_ state was modeled by superimposing native X-ray conformers of the Ni-F_430_ cofactor from cluster I templates (PDB: 1HBM, 3M32) onto our query structures. Thermodynamic analysis revealed a significant shift in cofactor affinity; the binding energy of Ni-F_430_ decreased from −305.64 kcal/mol in the Mcr_oxi-silent_ state −186.34 kcal/mol in the Mcr_silent_ receptor, providing the bioenergetic driving force for product release and subsequent enzyme reactivation. The docked pose of CoM-S-S-CoB exhibited high structural fidelity to the native crystal poses, achieving a favorable binding energy of −486.16 kcal/mol and a lowest CDOCKER score of −104.24 kcal/mol. Hotspot mapping of the active site was further utilized to identify the non-bonded interaction networks that stabilize the heterodisulfide. These findings suggest that the specific binding of CoM-S-S-CoB facilitates the induced-fit mechanism required to reset the catalytic pocket for subsequent substrate reduction.

### 4.3. Evaluating the Mutation Effects on the Binding Affinity Between Molecular Partners in the Query Mcr Receptor Complexes

To investigate the structural determinants of substrate selectivity and the energetic impact of active-site mutations in *M. ruminantium* Mcr, we performed in silico alanine-scanning mutagenesis. This approach was employed to evaluate the functional significance of 71 key residues per active site, utilizing the chemically inert nature of alanine to probe side-chain contributions to methanogenic bioactivity [58]. The relative binding affinity ($∆∆G_{mut}$) was quantified as the difference in binding free energy of the bound states ($∆G_{AB}$) and unbound ($∆G_{A-Bseperated}$) states to all ligands and between the alanine mutants and the wild-type (WT) enzyme in various oxidation states (Mcr_oxi-silent_ and Mcr_silent_). The binding free energy was defined as:

$$∆∆G_{mut}=∆∆G_{bind}^{\left(mutant\right)}-∆∆G_{bind}^{\left(wild\;type\right)}$$

$$AB↔A+B,∆G_{bind}=∆G_{AB}-∆G_{A-Bseperated}$$

where $∆G_{AB}$ represent the free energy of the ligand bound complex and $∆G_{A-Bseperated}$ represents the sum of the free energies of the isolated partners.

The total free energy ($∆G_{tot}$) was calculated as an empirical weighted sum of physical interactions:

$$∆G_{tot}\left(T\right)=aE_{vdw}+b∆G_{elec}\left(T\right)-c{TS}_{sc}+∆G_{np}$$

where *E_vdw_*, $∆G_{elec}$, *TS_sc_*, and $∆G_{np}$ represent van der Waals forces, electrostatic interactions, side-chain entropy, and non-polar solvation energy, respectively. The mutation energy function also contained entropy terms of the Mcr receptor’s side chain and protein backbone, which were evaluated according to room temperature in an aqueous solvent (when T is 273 K and the solvent dielectric constant is 80). Scaling factors were set to a = b = 0.5 and c = 0.8 based on optimized parameters for Mcr–ligand systems. Mutation resulting in $∆∆G_{mut}$ > 0.5 kcal/mol were classed as destabilizing, while those with $∆∆G_{mut}$ < −0.5 kcal/mol were considered stabilizing, enhancing substrate specificity for HS-CoM and CoB-SH (summarized in Tables S3 and S4). These energetic profiles were subsequently integrated into pharmacophore models for virtual screening of the PubChem database to identify high potency Mcr inhibitors.

### 4.4. Creating Pharmacophore Features of HS-CoM and CoB-SH Substrates on the Mcr Receptor Complex Structures with the Ni-F_430_ Cofactor

To elucidate the spatial and chemical requirements for *M. ruminantium* Mcr activity, we generated 3D interaction pharmacophore ensembles derived from the binding poses of HS-CoM and CoB-SH substrates (for Mcr_oxi-silent_) and the CoM-S-S-CoB product (for Mcr_silent_). Using an interaction pharmacophore generation protocol [59], we identified essential chemical features within a 5 Å radius of the docked ligands (demonstrated in Figure S9). For the Mcr_oxi-silent_ state, the top-ranked models consistently featured an eight-point pharmacophore (AAADDNNN: three H-bond acceptors, two H-bond donors, and three negative ionizable groups in Figure 7A). These models were scored using a Genetic Function Approximation (GFA) algorithm, yielding a consistent selectivity score of −4.6275. Similarly, the Mcr_silent_ state, representing the post-catalytic heterodisulfide binding, was characterized by an eight-point model (AAAADDNN) with a selectivity score of −3.8493 in Figure 7B. To enhance model specificity and prevent steric clashes, excluded volume constraints were integrated based on the coordinates of 71 key active-site residues (in Table 1). A minimum distance cutoff of 4 Å was enforced between any pharmacophore feature and the nearest receptor atom, ensuring the exclusion of sterically incompatible candidates during subsequent molecular screening.

## 5. Conclusions

Methyl-coenzyme M reductase (Mcr) is the metabolic engine of methanogenesis, catalyzing the final, rate-limiting reduction of methyl-coenzyme M and coenzyme B. Despite its critical role in enteric methane emissions, a primary driver of anthropogenic greenhouse gases, the structural architecture of Mcr from *Methanobrevibacter ruminantium*, the predominant genus (63.2%) in the bovine rumen, has remained experimentally elusive. In the study, we bridged this knowledge gap by generating a high-resolution structural model of the *M. ruminantium* Mcr heterohexamer (α_2_β_2_γ_2_, 269.9 kDa). Validated against multiple templates with 99% sequence similarity and robust stereochemical statistics (96.7% favored region occupancy), this model provides the first structural blueprint for this paramount enzyme in its native rumen context.

Beyond static topology, our integrated computational framework combining CDOCKER docking and alanine-scanning mutagenesis within the CHARMm force field elucidated the dynamic induced-fit mechanisms governing the Mcr catalytic cycle. We pinpointed a specialized ensemble of key residues (including Mcrα subunits of Arg270, Tyr332, Mcrβ Tyr367, and Mcr α′ Arg225) that dictate high-affinity substrate recognition and facilitate the transition across different oxidation states. These structural and energetic insights were distilled into two distinct 3D pharmacophore models: AAADDNNN for the Mcr_oxi-silent_ state and AAAADDNN for the heterodisulfide-bound Mcr_silent_ state (in Figure 7). These models represent a fundamental shift in our ability to quantify the physicochemical requirements of the Mcr active site, allowing for the precise targeting of its buried catalytic pocket.

The wealth of molecular and mechanistic information uncovered here serves as a transformative starting point for rational design of methanogen-specific inhibitors. By exploiting the volumetric constraints and unique pharmacophoric features of the *M. ruminantium* Mcr enzyme, future virtual screening efforts can identify small molecules with broad-spectrum efficacy across diverse rumen methanogens. Such precision chemogenetic interventions are essential for mitigating enteric methane emissions without compromising the metabolic health of the host, offering a scalable, science-driven strategy to address the global environmental challenges of livestock production.

## Figures and Tables

**Table 1:** Molecular interactions at the binding interfaces (5.0 Å) of the *M. ruminantium* Mcr (αβγ)_2_ complex with cofactor F_430_ and substrates.

2D-Interaction Coloration	Interaction Counterparts in the Mcr_ox1-silent_
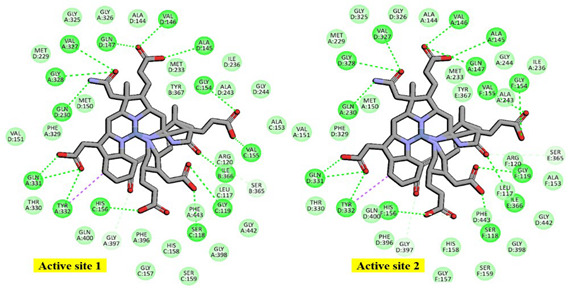	**F_430_ cofactor** **Active site 1**: (Mcrα) Gly325, Gly326, **Val327**, Gly328, **Phe329**, **Thr330**, **Gln331**, **Tyr332**, Phe396, Gly397, Gly398, Gln400, Gly442, **Phe443** (Mcrβ) Ser365, **Ile366**, **Tyr367** (Mcrγ) Leu117, **Ser118**, **Gly119**, **Arg120**, Ala153, Gly154, **Val155**, **His156**, Gly157, His158, Ser159 (Mcrα′) Ala144, **Ala145**, **Val146**, **Gln147**, Met150, Val151, Met229, **Gln230**, Met233, Ile236, **Ala243**, Gly244 **Active site 2:** (Mcrα) Ala144, Ala145, **Val146**, Gln147, Met150, Val151, Met229, **Gln230**, Met233, Gly244, ILE236, Ala243 (Mcrα′) Gly325, Gly326, **Val327**, **Gly328**, Phe329, Thr330, Gln331, Tyr332, Phe396, Gly397, Gly398, Gln400, Gly442, **Phe443** (Mcrβ′) Ser365, Ile366, **Tyr367** (Mcrγ′) Leu117, Ser118, Gly119, **Arg120**, Ala153, **Gly154**, Val155, His156, Gly157, His158, Ser159
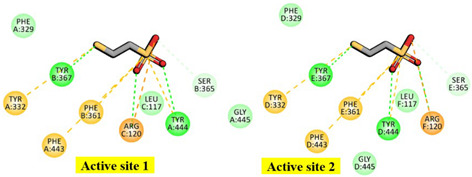	**CoM-SH** **Active site 1**: (Mcrα) Phe329, **Tyr332**, Phe443, **Tyr444**, **Gly445** (Mcrβ) **Phe361**, Ser365, **Tyr367** (Mcrγ) Leu117, **Arg120** **Active site 2:** (Mcrα′) Phe329, Tyr332, Phe443, Tyr444, Gly445 (Mcrβ′) Phe361, Tyr367, Ser365 (Mcrγ′) Leu117, Arg120
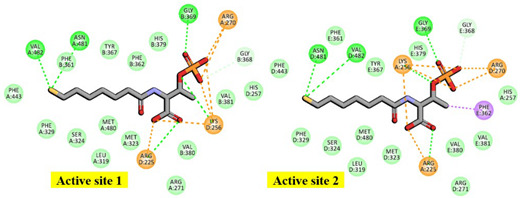	**CoB-SH** **Active site 1**: (Mcrα) **Arg270**, Arg271, Leu319, Met323, Ser324, **Phe329**, **Phe443**, Met480, **Asn481**, **Val482** (Mcrβ) **Phe361**, **Phe362**, **Tyr367**, **Gly368**, **Gly369**, **His379**, Val380, Val381 (Mcrα′) **Arg225**, **Lys256**, **His257** **Active site 2**: (Mcrα) Arg225, Lys256, His257 (Mcrα′) Arg270, Arg271, Leu319, Met323, Ser324, Phe329, Phe443, Met480, Asn481, Val482 (Mcrβ′) Phe361, Phe362, Tyr367, Gly368, Gly369, His379, Val380, Val381

**Table 2:** Molecular interactions at the binding interfaces (5.0 Å) of the *M. ruminantium* Mcr (αβγ)_2_ complex with F_430_ and the heterodisulfide product CoM-S-S-CoB.

2D-Interaction Coloration	Interaction Counterparts in the Mcr_silent_ *
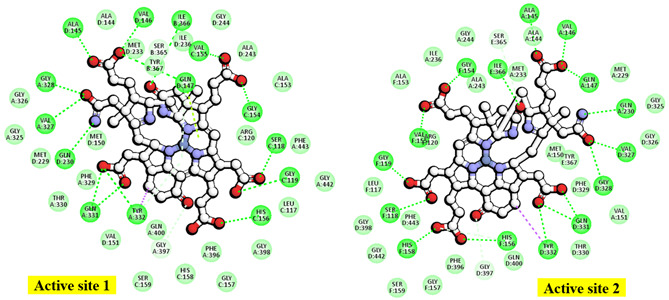	**F_430_ cofactor** **Active site 1**: (Mcrα) Gly325, Gly326, Val327, Gly328, **Phe329**, Thr330, Gln331, **Tyr332**, Phe396, Gly397, Gly398, Gln400, Gly442, **Phe443** (Mcrβ) Ser365, Ile366, **Tyr367** (Mcrγ) Leu117, Ser118, Gly119, Arg120, Ala153, Gly154, Val155, His156, Gly157, His158, Ser159 (Mcrα′) Ala144, Ala145, Val146, **Gln147**, Met150, Val151, Met229, Gln230, Ala243, Met233, Ile236, Gly244 **Active site 2**: (Mcrα) Ala144, Ala145, Val146, **Gln147**, Met150, Val151, Met229, Gln230, Met233, Ile236, Ala243, Gly244 (Mcrα′) Gly325, Gly326, Val327, Gly328, Phe329, Thr330, Gln331, Tyr332, Phe396, Gly397, Gly398, Gln400, Gly442, Phe443 (Mcrβ′) Ser365, Ile366, Tyr367 (Mcrγ′) Leu117, Ser118, Gly119, Arg120, Ala153, Gly154, Val155, His156, Gly157, His158, Ser159
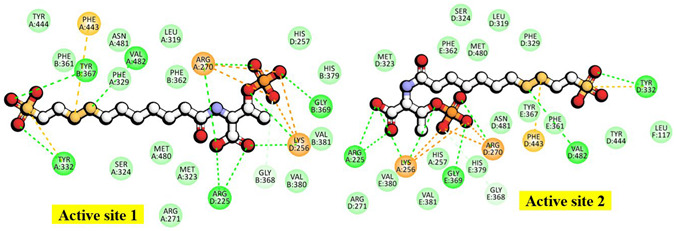	**CoM-S-S-CoB** **Active site 1**: (Mcrα) Arg270, Arg271, Leu319, Met323, Ser324, **Phe329**, **Tyr332**, **Phe443**, Tyr444, Met480, **Asn481**, **Val482** (Mcrβ) **Phe361**, Phe362, **Tyr367**, **Gly368**, **Gly369**, His379, Val380, Val381 (Mcrα′) Arg225, Lys256, His257 **Active site 2**: (Mcrα) Arg225, Lys256, His257 (Mcrα′) Arg270, Arg271, Leu319, Met323, Ser324, Phe329, Tyr332, Phe443, Tyr444, Met480, Asn481, Val482 (Mcrβ′) Phe361, Phe362, Tyr367, Gly368, Gly369, His379, Val380, Val381 (Mcrγ′) Leu117

* The coenzymes CoM-SH and CoB-SH, which bind separately in the Mcr_oxi-silent_ state, undergo covalent coupling to form a heterodisulfide in the Mcr_silient_ state.

**Table 3:** Decomposition of binding free energy components between the *M. ruminantium* reductase and non-bonded ligands during sequential docking to the Ni-F_430_ cofactor. All values are reported in kcal/mol.

Ligand Name	Binding Energy	Ligand Energy	Protein Energy	Complex Energy	Entropic Energy	Complex Entropy	Protein Entropy	Ligand Entropy
F_430_ cofactor (Mcr_oxi-silent_)	−305.64	591.78	321.87	322.16	22.02	−36.75	−35.75	−22.03
CoM-SH (Mcr_oxi-silent_)	−19.06	0.60	321.87	321.85	17.02	−35.75	−35.75	−17.02
CoB-SH (Mcr_oxi-silent_)	−804.56	43.49	321.87	321.11	19.69	−35.75	−35.75	−19.68
F_430_ cofactor (Mcr_silent_ state_)_	−186.34	93.51	322.36	322.26	21.96	−35.76	−35.75	−21.97
CoM-S-S-CoB (Mcr_silent_ state)	−486.16	−120.13	322.36	321.75	21.06	−35.78	−35.75	−21.06

Note: Methane bioengenesis is catalyzed by the methyl-coenzyme M reductase (Mcr), utilizing CoM-SH and CoB-SH as co-substrates. Notably, Mcr is highly sensitive to oxidative inactivation. The thermodynamic stability of the complex was evaluated using the Gibbs free energy of binding, defined as **Δ*G_binding_* = Δ*G_complex_* − (Δ*G_protein_* + Δ*G_ligand_*)**, where the total free energy is derived from the fundamental relation **Δ*G* = Δ*H* − *T*Δ*S***.

## Data Availability

Beyond the complex structures available as Supplementary materials, the pharmacophore data for each oxidation state may be requested from the corresponding author (Han-Ha Chai). Please note that these data are not currently accessible through public repositories, as the raw and processed data are integral to an ongoing study and cannot be fully shared at this stage.

## Supplementary Materials

The following supporting information can be downloaded at: https://www.mdpi.com/article/10.3390/ijms27020995/s1. References [60,61,62,63] are cited in the Supplementary Materials.

## Abbreviations

The following abbreviations are used in this manuscript:

*M. ruminantium*	*Methanobrevibacter ruminantium*
Mcr	Methyl-coenzyme M reductase
Mcr_oxi-silent_	Reduced Mcr bound to HS-CoM and CoB-SH inactive state
Mcr_silent_	Oxidized Mcr bound to the heterodisulfide CoM-S-S CoB inactive state
VFAs	Volatile Fatty Acids
CH_3_-S-CoM	Methyl-Coenzyme M
HS-CoM	Coenzyme M
CoB-SH	Coenzyme B
Hdr	Heterodisulfide reductase
PME	Particle Mesh Ewald
DOPE	Discrete Optimized Protein Energy score
PDF	Probability Density Function energy
WT	Wild-Type enzyme
GFA	Genetic Function Approximation
GB	Generalized Born model
