# Supplementary material for: Uncovering Major Structural and Functional Features of Methyl-Coenzyme M Reductase (MCR) from Methanobrevibacter ruminantium in Complex with Two Substrates
Source: Int J Mol Sci. 2026 Jan 19;27(2):995. doi: 10.3390/ijms27020995 (PMC12842094; doi:10.3390/ijms27020995)
Supplement: Supplementary file 1 [file ijms-27-00995-s001.zip › ijms-4073770-supplementary.pdf]

1. Supplementary Materials

1.1. A structure model of methyl-coenzyme M reductase from *Methanobrevibacter ruminantium*

The protein sequences for the Mcr subunits (*mcrBDCGA*) of *M. ruminantium* were used as queries for ortholog retrieval via BLASTP. Sequence alignments were performing using CLUSTALW to delineate conserved regions across cluster I methanogens (**Figs. S6-S8**). Sequence identifies between the query and templates were as follows: McrA (80.4%), McrC (77.8%), McrG (76.3%), McrB (76.1%), and McrD (47.7%). The high similarity in the McrABG core subunits supports the structural relevance of the $(\alpha\beta\gamma)_2$ heterohexameric assembly. Among the 29 available Mcr crystal structures, 18 belong to cluster I. We prioritized five templates with resolutions between 1.1-1.19 Å for high-fidelity modeling: (1) $Mcr_{oxi\text{-}silent}$ State: PDB IDs 1HBN, 5A0Y, and 5G0R. These capture the enzyme bound with the Ni-F_{430} cofactor and both HS-CoM and CoB-SH coenzymes. (2) Mcr_{silent} State: PDB ID 1HBM. This structure represents the enzyme bound with the heterodisulfide product, CoM-S-S-CoB. Structural pair alignments were conducted using the iFATCAT server, yielding an RMSD of 0.32-0.52Å over 2,470 equivalent positions (**Fig. S9** and **Fig.S10**). The query sequences were mapped onto the conserved core framework of the (αβγ) monomer folds from *M. thermautotrophicus* and *M. marburgensis.* The pseudo-symmetry axis between Mcrα and Mcrβ subunits was utilized to reconstruct the tetrameric $(\alpha\beta)_2$ core, followed by the integration of Mcrγ subunits to complete the heterohexamer. Special attention was paid to the substrate channel, ensuring that all ligands were fully buried in a manner consistent with experimental ligandation patterns observed in the templates.

The *M. ruminantium* Mcr model was built by replacing backbone atoms of the query sequence at aligned positions of the selected structural templates. Because the Mcrγ subunit lacks intrinsic symmetry, two symmetry-related units (γ and γ′) were manually docked onto the tetrameric $Mcr(\alpha\beta)_2$ core at positions corresponding to the template architecture. Threading was performed for each subunit, evaluating the target-template fit through a sequence to structure alignment of 2,476 residues. Genomic optimizing was conducted through several rounds of restrained molecular dynamics and simulated annealing. (1) Minimization: bad contacts were resolved using geometry optimization with the PME option, resulting in an energy of -388.578 kcal/mol. (2) Scoring: the best-fit model was selected based on a PDF energy of -176,390.7 kcal/mol (geometric restraints) and a DOPE score of -288,498.8 kcal/mol (relative conformational stability). (3) Refinement: Loop and side-chain refinement were focused on 42 critical residues (1.70% of the total structure) that exhibited local instability or poor threading scores. The final model spans from Mcrα (Ala2-Ala549), Mcrβ (Ala2-Ile443), to Mcrγ (Ala2-Glu249). The internal 33Å methane transport channel, defined by specific segments in the Mcrα subunit (Pro330-Leu360, Gln400-Tyr446, Val482-Ile491), was validated for continuity. This channel connects the buried Ni-F_{430} cavity to the enzyme surface, maintaining the conserved funnel-like geometry observed in *M. marburgensis* (PDB: 3M1V).

Validation of the final *M. ruminantium* Mcr structure was performed using the PROCHCK algorithm. The distribution of (Φ, Ψ) dihedral angles showed that 96.7% of residues were positioned within the core favored regions, with the remaining residues primarily located in non-critical loop segments. This score is marginally lower than the 97.0% observed in the five experimental templates (**Table S1**), a discrepancy attributed to the absence of crystal packing constraints and solvent-stabilization effects in the computational model. A comparative analysis between *M. ruminantium* query and templates (*M. marburgensis* and *M. thermautotrophicus*) revealed differences in surface ion sequestration. (1) Template Structures: Exhibit high occupancy of Mg^{2+}, Na^{+}, and Cl^{-} ions, which neutralize acidic side-chains (predominantly glutamate) to prevent electrostatic repulsion and protein aggregation during X-ray crystallization. (2) Query Model: Optimized in a solvent-free or implicit solvent environment, the query model does not include coordinates cations. Consequently, the surface acidic clusters exhibit higher flexibility. These differences align with the mesophilic nature

to *M. ruminantium*, whose habitat in the rumen intestinal tract mimics the physiological traits of *M. marburgensis* rather than the halophilic or thermophilic adaptations requiring extensive ion-binding for structural rigidity. The flexibility observed in our model may represent a more biologically relevant state for substrate-induced fit during active methanogenesis.

1.2. Complex models of the query Mcr receptor with F_{430} and substrates in two different enzyme states

The active site geometry was defined based on conserved features, the H-bond network, hydrophobic annulus, and the glycine-rich loop. Docking spheres were centered at the following coordinates derived from the F_{430} and coenzyme binding cavities: (1) Ni-F_{430} center: (15.68, 44.45, -31.50) with a radius of 35.77Å, (2) CoB-SH Binding Site: (22.42, 37.11, -17.42) with a radius of 8.40Å, these coordinates ensured that the ligands were fully encapsulated within the 30Å long substrate channel [1–3]. Rigid-body superimposition was followed by soft-body docking to accommodate the "movable lands" of the active site. (1) Simulation Parameter: Random orientations were generated via translation to the specified hotspots. Simulated annealing involved 2,000 heating steps to 700K, followed by 5,000 cooling step to 300K. (2) Scoring: the top 10 poses for each substrate were ranked based on the sum of interaction energy and ligand internal strain. The lowest (most negative) scores were retained as the bioactive conformers. The binding energy (ΔE_{bind}) was calculated using the equation: $\Delta E_{Binding} = E_{Complex} - (E_{Ligand} + E_{Receptor})$. Calculation utilized statistical mechanics within the CHARMm force field at an entropy temperature of 298.15K. Principal moments of inertia were converted into rotational and translational entropies, assuming a symmetry value (σ) of 1 for non-symmetric molecules [62]. Decomposed components of the free energy for the *M. ruminantium* $Mcr_{oxi-silent}$ state including van der Waals and electrostatic contributions are summarized in **Table 3**.

Conformational changes induced prior to and during heterodisulfide formation were monitored by aligning the *M. ruminantium* model with known product-bound states. The specific active site spheres used for docking and hotspot calculation were defined as follows: (1) $Mcr_{oxi-silent}$ Transition Spheres: (35.21, 35.82, -58.89) with a radius of 9.22Å. (2) Mcr_{silent} Active Site Center: (26.59, 38.24, -50.11) with a radius of 11.40Å. The heterodisulfide product CoM-S-S-CoB was docked using CDOCKER protocol. The reliability of the docking poses was assessed through a hierarchical scoring approach: (1) $Mcr_{oxi-silent}$ State: The lowest CDOCKER score of -280.57 kcal/mol was recorded, with a calculated binding energy of -186.34 kcal/mol, indicating sufficient potential to drive substrate relocation. (2) Mcr_{silent} State: The top-scoring pose (CDOCKER Score: -104.24 kcal/mol) demonstrated high congruence with native X-ray product conformers. The subsequent calculation of the binding energy for the CoM-S-S-COB product yield -486.16kcal/mol, facilitating a detailed interpretation of the hydrophobic and electrostatic interaction interfaces (**Fig. S12**).

1.3. Evaluating the mutation effects on the binding affinity between molecular partners in the query Mcr receptor complexes

All energetic terms were computed using the CHARMm force field applied to modeled protein structures of *M. ruminantium* Mcr. Electrostatic contributions (ΔG_{elec}) were evaluated using the Generalized Born (GB) implicit solvent model [63]. To account for the aqueous environment, calculations were performed at a reference temperature of 273K with a solvent dielectric constant (ε) of 80. The mutation energy function explicitly incorporated entropy terms for the Mcr receptor's side chains and backbone to account for conformational flexibility upon ligand binding. For the wild-type reference, the model primarily focused on electrostatic contributions, whereas for alanine variants, the full suite of non-electrostatic terms (including the cavitation energy, ΔG_{np} [60]) was integrated to capture the total energetic shift. Scaling factors for alanine variants of the 62 key residues (detained in **Table 2**) were kept consistent with the pH-independent mutation energy protocols established for the Ni-F_{430} cofactor and the CoM-S-S-CoB product. Detailed summaries of

mutation energies and structural stability relationships for the various Ni-Mcr oxidation states are provided in **Tables S3-S6**. These tables delineate the physicochemical transitions between $Mcr_{oxi\text{-}silent}$ and Mcr_{silent} states, providing the basis for understanding the catalytic transformation of substrates above the F_{430} cofactor.

1.4. Creating pharmacophore features of HS-CoM and CoB-SH substrates on the Mcr receptor complex structures with the Ni-F_{430} cofactor

The pharmacophore ensembles were constructed to capture the precise 3D locations and directional constraints of chemical substructures essential for methanogenic catalysis. The interfeature spacing was constrained to a minimum of 2Å to ensure distinct spatial resolution. For each binding site on the hexadimeric $Mcr_{oxi\text{-}silent}$ structure, we retained the 10 highest-scoring models, with each ensemble required to possess between four and eight features. To refine the chemical space of the active site, we implemented excluded volume spheres representing the 71 key residues identified in the Mcr receptor (**Table 1**). These shapes acted as physical barriers, mimicking the steric environment of the channel apex where the Ni-F_{430} cofactor residues. This arrangement specifically suppressed potential steric clashes for ligands fitting onto the Ni-F_{430} cofactor. The transition from the $Mcr_{oxi\text{-}silent}$ (substrate-bound) to the Mcr_{silent} (product-bound) state was mapped by contrasting the spatial arrangement of chemical probes. While the $Mcr_{oxi\text{-}silent}$ features represented non-covalent contacts of HS-CoM and CoB-SH, the Mcr_{silent} models reflected the covalent connectivity of the CoM-S-S-CoB heterodisulfide following nucleophilic attack at the Ni-F_{430} center (**Fig. 3**). The selectivity scores and selectivity (the capacity to distinguish active from inactive compounds) were evaluated using the GFA model. We acknowledge that the limited availability of experimentally verified inactive molecules for the Mcr receptor may influence the absolute sensitivity values; however, the high selectivity scores obtained (-4.6275 to -3.8493) indicate a robust framework for identifying potent inhibitors within the PubChem chemical libraries.

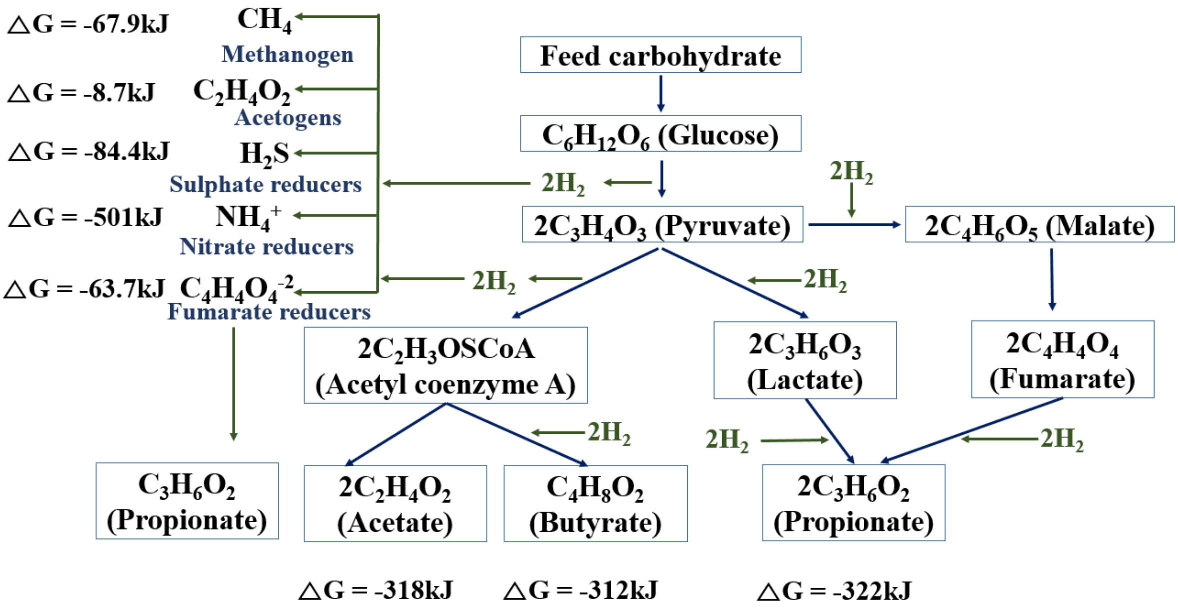

Figure S1. Schematic of the major pathway of rumen fermentation with H_2 transfer and then estimated Gibbs free energies without considering ATP generation [7], where generation and incorporation of H_2 are estimated based on 1mol of glucose fermentation according to the following reactions: $C_6H_{12}O_6$ (glucose) → $2C_3H_4O_3$(pyruvate) + $2H_2$; $2C_3H_4O_3$ + 2HSCoA(non-esterified

coenzyme A) → $2C_2H_3OSCoA$(acetyl coenzyme A) + $2CO_2$ + $2H_2$; C_2H_3OSCoA + H_2O(water) → $C_2H_4O_2$(acetate) + HSCoA; $2C_2H_3OSCoA$ + $2H_2$ → $C_4H_8O_2$(butyrate) + 2HSCoA; $2C_3H_4O_3$ + $2H_2$ → $2C_3H_6O_3$(lactate); $2C_3H_6O_3$ + $2H_2$→ $2C_3H_6O_2$(propionate) + $2H_2O$; $2C_3H_4O_3$ + $2H_2$ + $2CO_2$ (carbon dioxide) → $2C_4H_6O_5$(malate); $2C_4H_4O_4$(fumarate) + $2H_2$ → $2C_3H_6O_2$ + $2CO_2$. The H_2 concentration in the rumen fluid is near 1μM. A small increase in the H_2 concentration leads to both down-regulation of H_2 generating pathway and up- regulation of H_2-natural and H_2-consuming pathways such as propionate formation, resulting in additional energy supply to host ruminants, in contrast to the generation of CH_4 by methanogenic archaea where up to 12% of the gross energy ingested by host ruminants is lost [61].

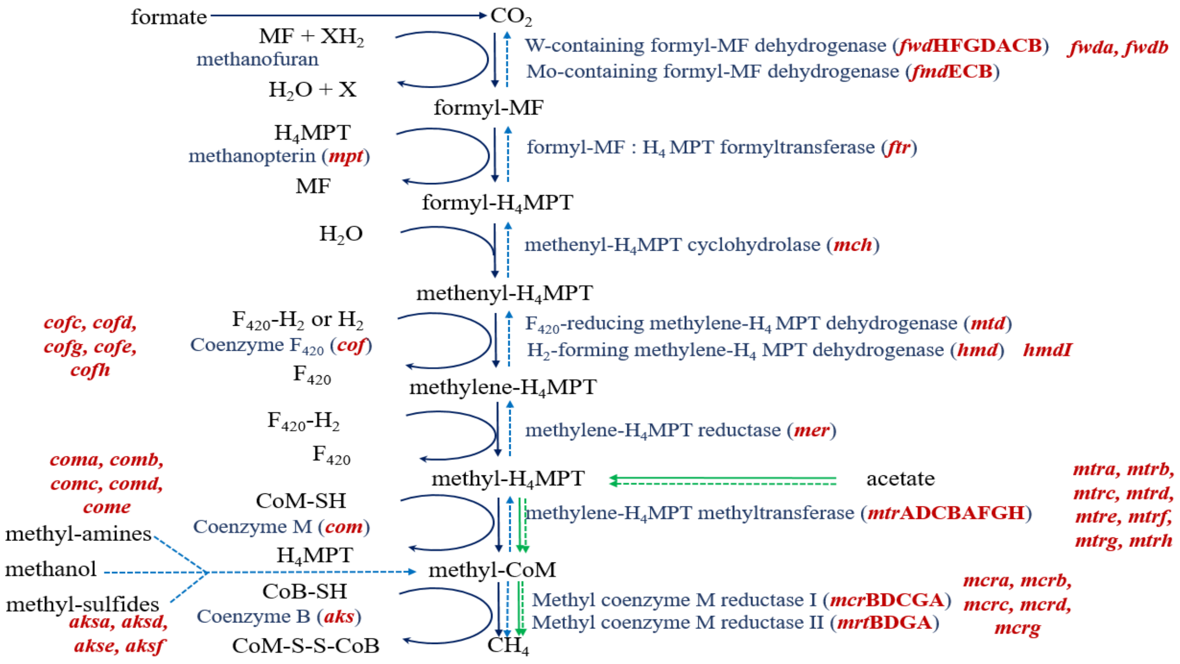

Figure S2. Overview of methanogenesis pathways and the central role of methyl-coenzyme M. The metabolic flux is depicted for hydrophobic (solid blue arrows), methylotrophic (broken blue arrows), and acetoclastic (double-lined green arrows) pathways. Methanogenesis is orchestrated by at least 16 essential coding genes (*cof*D, *fwd*A, *hmd*I, *mtr*A, *mtr*B, *mtr*C, *mtr*D, *mtr*E, *mtr*F, *mtr*G, *mtr*H, *mcr*A, *mcr*B, *mcr*C, *mcr*D, and *mcr*G [29]) In the hydrophobic route, the CO_2-derived formyl group undergoes a six-step reduction to a methyl group via the tetrahydromethanopterin (H_4MPT) carrier [48]. Subsequently, the methyl group is transferred from methyl- H_4MPT to coenzyme M (HS-CoM), yielding methyl-coenzyme M (CH_3-S-CoM), the universal intermediate for all methanogenic pathways. The final reduction of CH_3-S-CoM to methane is coupled with the generation of an electrochemical proton (H^+) gradient, driving ATP synthesis. Collectively, these biological processes account for approximately 70% of global methane emissions.

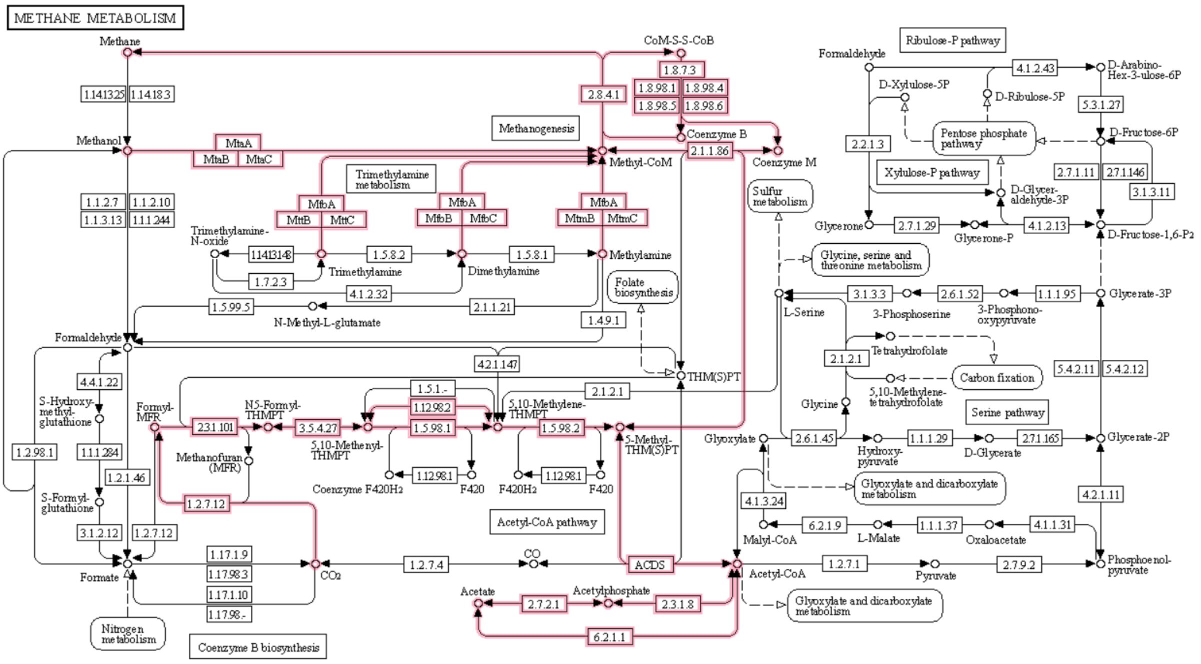

Figure S3. Global metabolic landscape of methanogenesis based on KEGG pathway analysis. The schematic integrates four primary methanogenic routes: hydrogenotrophic (M00567), acetoclastic (M00357), and methylotrophic pathways utilizing methanol (M00356) or methylamine (M00563). Major flux directions for the reduction of CO_2, acetate, methanol, and methylamine to methane are indicated by thick arrows. Key enzymes and intermediate substrates in each pathway are highlighted in pink. In the ruminant gastrointestinal tract, methanogenic archaea occupy the terminal niche of the trophic chain, primary utilizing CO_2 and H_2 to generate methane. The process serves as the critical H_2 sink, maintaining rumen fluid [61] H_2 concentration at approximately 1μM to facilitate optimal fermentation energetics.

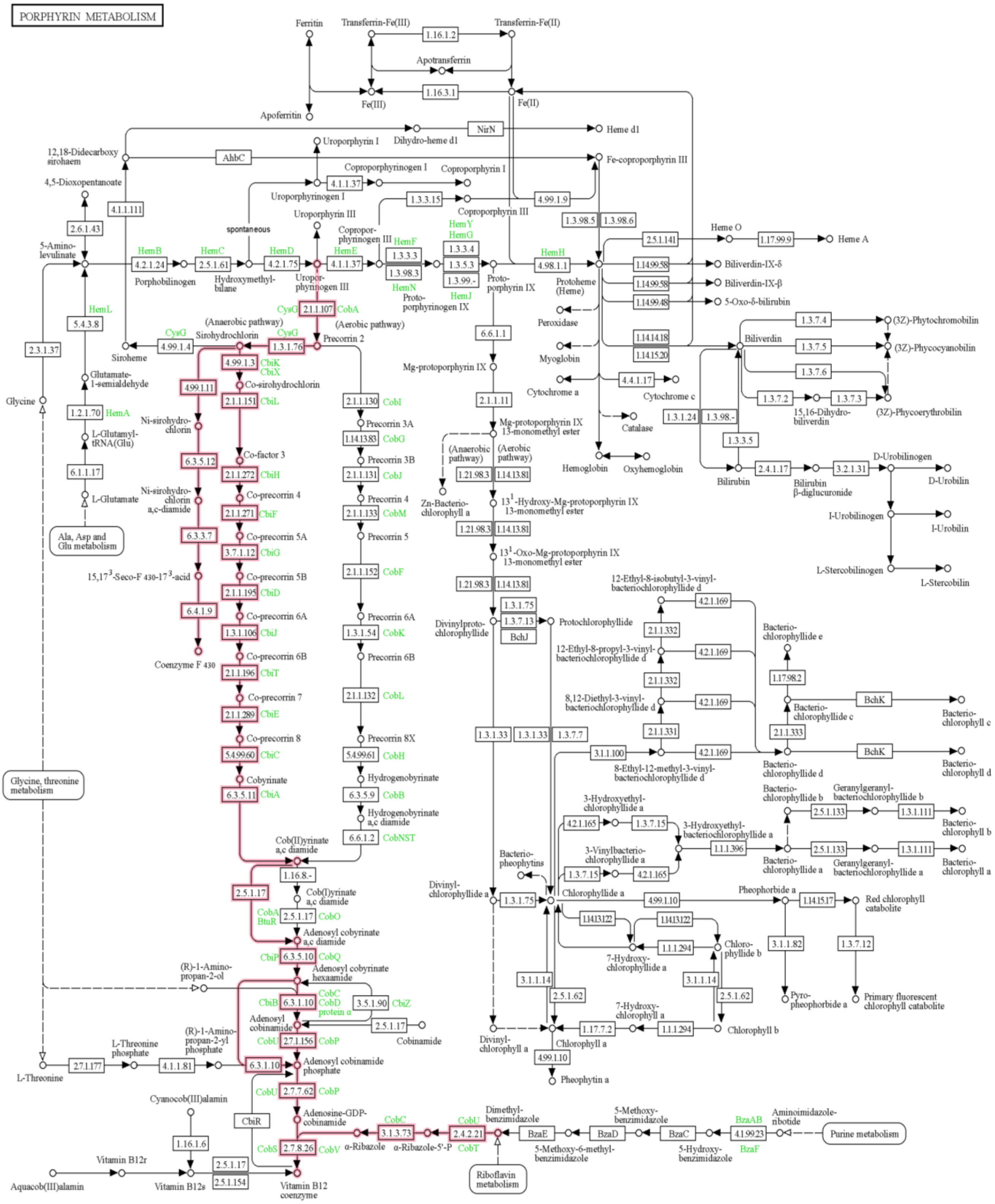

Figure S4. Anaerobic biosynthesis of vitamin B_{12} and its regulatory role in methanogen-associated hydrogen utilization. The schematic illustrates the anaerobic biosynthetic pathway of cyanocobalamin (vitamin B_{12}) within the framework of porphyrin metabolism in methanogenic archaea. Vitamin B_{12} serve as a critical cofactor in the propionate ($C_3H_6O_2$) production pathway. By modulating the flux toward propionate synthesis, vitamin B_{12} plays a pivotal role in regulating H_2 utilization, effectively competing for reducing equivalents otherwise used in methanogenesis. As illustrated in **Fig. S1**, this metabolic redirection represents a strategic pathway for the mitigation of biological methane emissions.

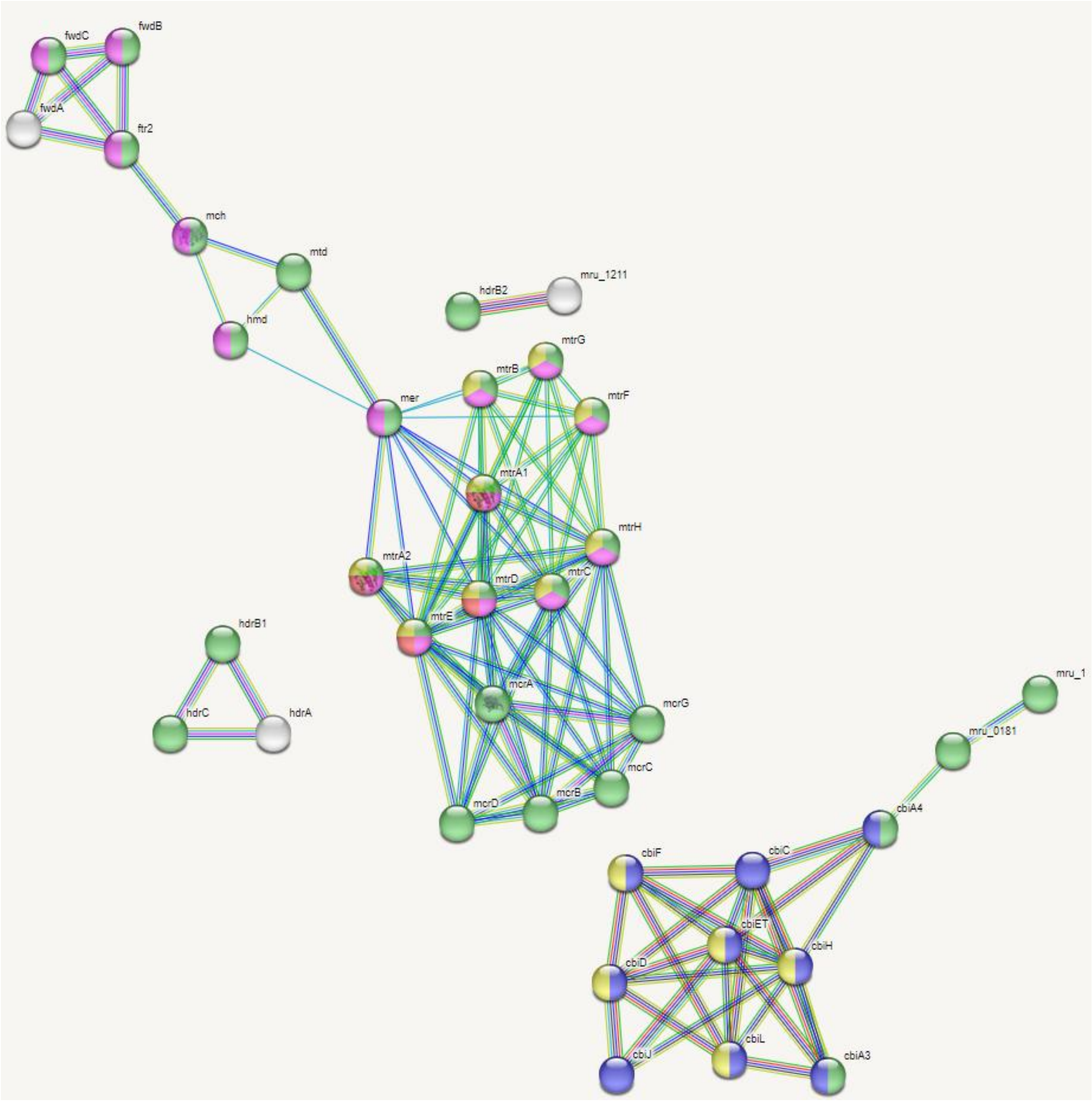

Figure S5. Genomic core architecture and metabolic module of methanogenesis in *M. ruminantium*. The methanogenic machinery is organized into three primary functional gene clusters. (i) Hydrogenotrophic pathway modules: This core includes essential enzymes for CO_2 reduction and methyl-transfer, such as *ftr*, *fwd*ABC, *mch*, *mtd*, *hmd*, *mer*, and *mcr*ABCDG operon (Mcr isoenayme I). A key step is mediated by the methyltransferase complex (mtrABCDFGH), structured as a tetrameric heterooctamer [mtr(A-H)$_4$], which catalyze the transfer of the methyl moiety to HS-CoM (CH_3-H_4MPT + HS-CoM $\rightarrow$ H_4MPT + CH_3-S-CoM), (ii) cofactor and coenzyme biosynthetic loci: These genes facilitates the anaerobic synthesis of essential cofactors, notably the *cbi*ACDFHJL cluster within the porphyrin metabolic pathway, governing the transition from uroporphyrin III to cobyrinate (**Fig. S4).** (iii) Heterodisulfide salvage system: The *hdr*ABC complex mediates the final regeneration of coenzymes via the reduction of the CoM-S-S-CoB heterodisulfide. As illustrated in **Fig. S1,** the Mcr complex serves as the universal catalytic engine across all methanogenic lineages, executing the terminal step of CH_4 biogenesis.

.

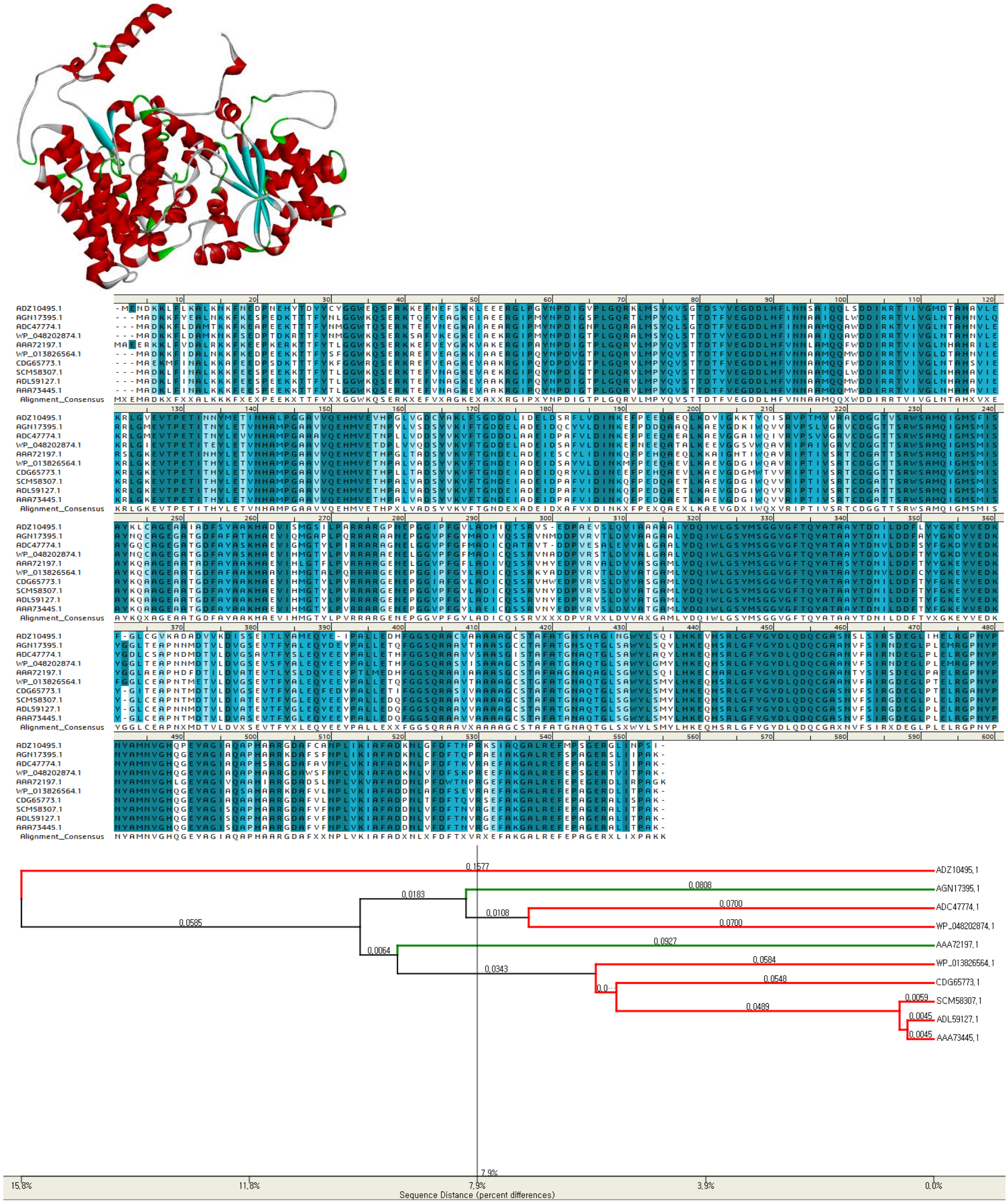

Figure S6. Structural model and conserved sequence architecture of the *M. ruminantium* McrA subunit.

The homology model of McrA subunit (~65kDa, 550 amino acids) from *Methanobrevibacter ruminantium* was constructed based on high-fidelity sequence alignments. Structural trace residues between the query and templates were identified within a specified distance cutoff of 7.9%, situating the query within the phylogenetic cluster I [24]. A comparative analysis was performed using nine representative McrA template sequences: *Methanobacterium lacus* (ADZ10495.1), *Methanobrevibacter sp. AbM4* (AGN17395.1), *Methanobrevibacter smithii* (WP_048202874.1), *Methanothermus fervidus* (AAA72197.1), *Methanobacterium paludis* (WP_013826564.1), *Methanobacterium sp. MB1* (CDG65773.1), *Methanothermobacter wolfeii* (SCM58307.1), *Methanothermobacter marburgensis str. Marbug* (ADL59127.1), and *Methanothermobacter thermautotrophicus* (AAA73445.1). The *M. ruminantium* McrA query (ADC47774.1) exhibits robust structural conservation with these templates, ensuring the reliability of the resulting fold model.

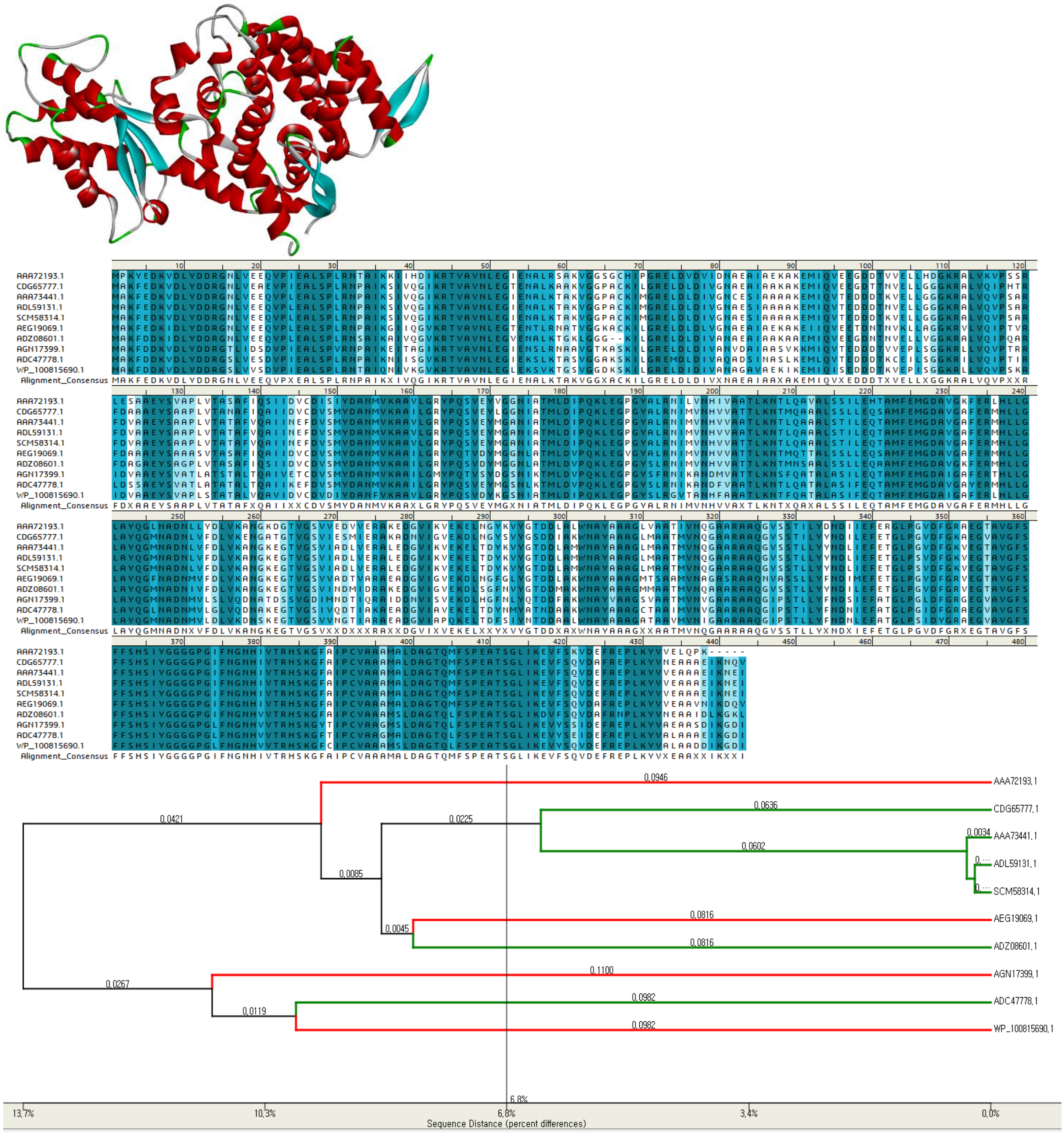

Figure S7. Structural modeling and comparative sequence analysis of the *M. ruminantium* McrB subunit.

The homology-based fold model of the McrB subunit (~45kDa, 443 amino acids) from *Methanobrevibacter ruminantium* was generated using high-resolution templates. Structural trace residues between the query and template sequences were aligned within a stringent distance cutoff of 6.8%, confirming its placement within the phylogenetic cluster I. A comprehensive comparative analysis was conducted using nine representative McrB templates: *Methanothermus fervidus* (AAA72193.1), *Methanobacterium sp. MB1* (CDG65777.1), *Methanothermobacter thermautotrophicus* (AAA73441.1), *Methanothermobacter marburgensis str. Marburg* (ADL59131.1), *Methanothermobacter wolfeii* (SCM58314.1), *Methanobacterium paludis* (AEG19069.1), *Methanobacterium lacus* (ADZ08601.1), *Methanobrevibacter sp. AbM4* (AGN17399.1), and *Methanobrevibacter smithii* (WP_100815690.1). the *M. ruminantium* McrB query (WP_100815690.1) exhibits high structural homology and sequence conservation across these phylogenetically diverse templates.

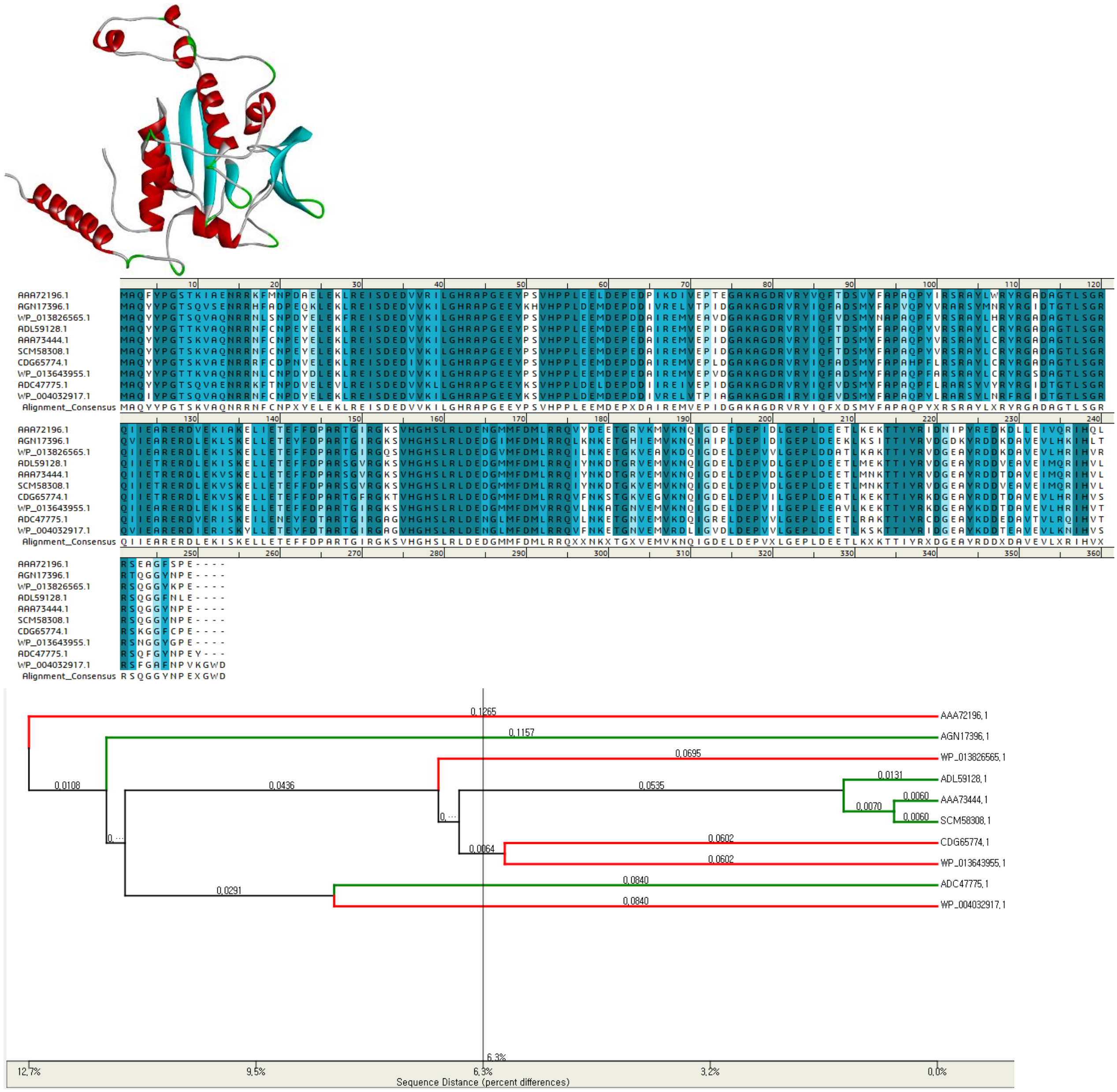

Figure S8. Structural modeling and comparative sequence analysis of the *M. ruminantium* McrG subunit.

The homology-based fold model of the McrG subunit (~35kDa, 250 amino acids) from *Methanobrevibacter ruminantium* was constructed using high-resolution templates. Structural trace residues between the query and template sequences were aligned within a stringent distance cutoff of 6.3%, confirming its classification within phylogenetic cluster I. A comprehensive comparative analysis was performed using nine representative McrGn templates: *Methanothermus fervidus* (AAA72196.1), *Methanobrevibacter sp. AbM4* (AGN17396.1), *Methanobacterium paludis* (WP_013826565.1), *Methanothermobacter marburgensis str. Marburg* (ADL59128.1), *Methanothermobacter thermautotrophicus* (AAA73444.1), *Methanothermobacter wolfeii* (*SCM58308.1*), *Methanobacterium sp. MB1* (CDG65774.1), *Methanobacterium lacus* (WP_013643955.1), and *Methanobrevibacter smithii* (WP_004032917.1). The *M. ruminantium* McrG query (ADC4777.1) exhibits significant structural homology and sequence conservation across these phylogenetically diverse templates.

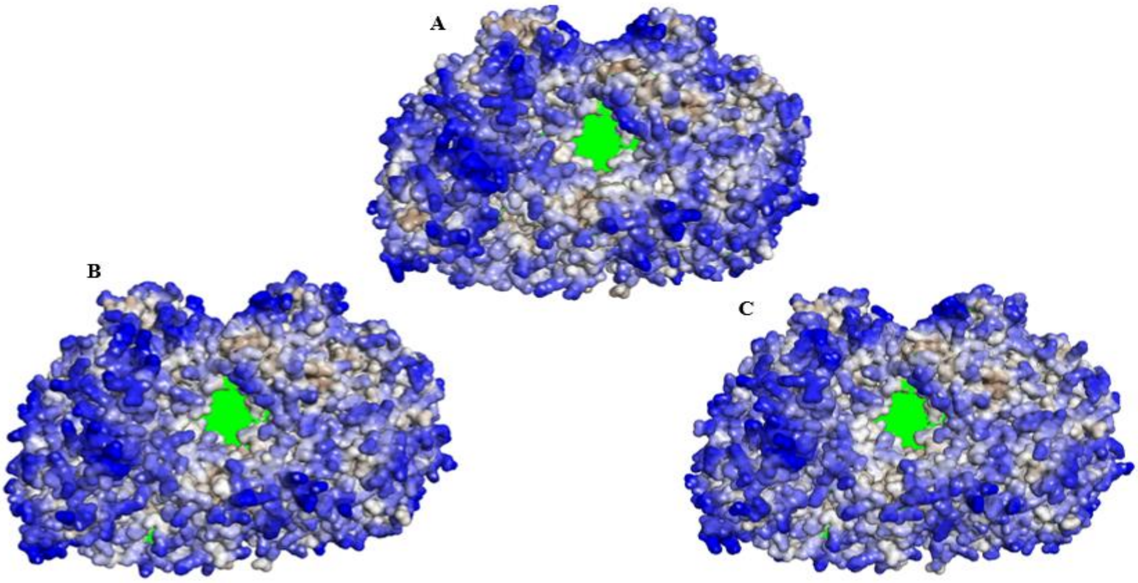

Figure S9. Comparative analysis of hydrophobic surface potentials across structurally characterized Mcr enzymes. Surface representations of (A) the *M. ruminantium* query model, (B) *M. thermautotrophicus* (1HBN) and (C) *M. marburgenesis* str. *Marbug* (5A0Y). The potential is mapped on a gradient from hydrophilic (blue, negative) to hydrophobic (brown, positive). Dark green regions delineate the entrance funnels for HS-CoM and CoB-SH to the catalytic center. Field-fit alignment was optimized by maximizing a weighted energy function (50% electrostatic and 50% steric contributions), yielding similarity scores of 0.9115 between templates (1HBN and 5A0Y) and 0.839 relative to the query. While all three enzymes exhibit strictly conserved hydrophobic cores within phylogenetic class I, structural divergence is localized to solvent-exposed loops (e.g. Mcrα 40 to 61, 357 to 368, Mcrβ 281 to 290, and Mcrγ 181 to 188). Notably, the C-terminal region of the Mcrγ subunit demonstrates differential interaction models with the α and β subunits. Pronounced variations in surface topography, loop geometry, and electrostatic profiles are observed primarily at the active site funnel.

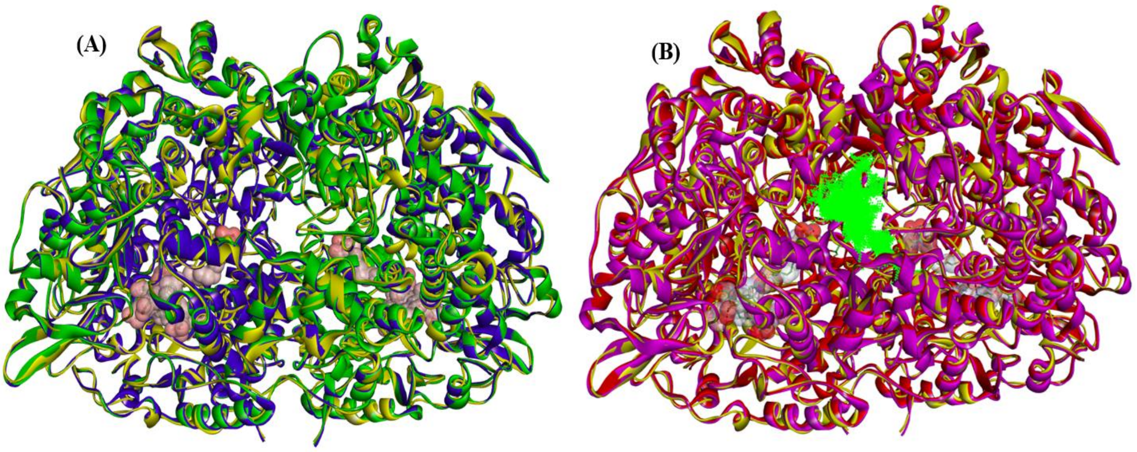

Figure S10. Structural superposition and substrate channel architecture of Mcr complexes. The *M. ruminantium* Mcr query model (yellow ribbon) is superposed with representative templates, demonstrating high structural conservation across both (A)$Mcr_{oxi-silent}$ and (B) Mcr_{silent} states. (A) $Mcr_{oxi-silent}$ configurations: *M. thermautotrophicus (*green; 1HBN) and *M. marburgenesis* str. *Marbug* (blue; 5A0Y). (B) Mcr_{silent} configuration: *M. thermautotrophicus* (pink; 1HBM) and *M. marburgenesis* str. *Marbug* (red; 3M32). The substrate channel aperture is delineated by green points. The two funnel-shaped channels extend 30 Å from the protein surface to the active site region, remaining 50 Å apart from two independent, structurally identical catalytic sites. Both coenzymes (HS-CoM

and CoB-SH) across the active site through a unified channel, which initiates with a 25 Å diameter at the surface and narrows to 8 Å over the final 16 Å leading to the F_{430} cofactor pocket [28].

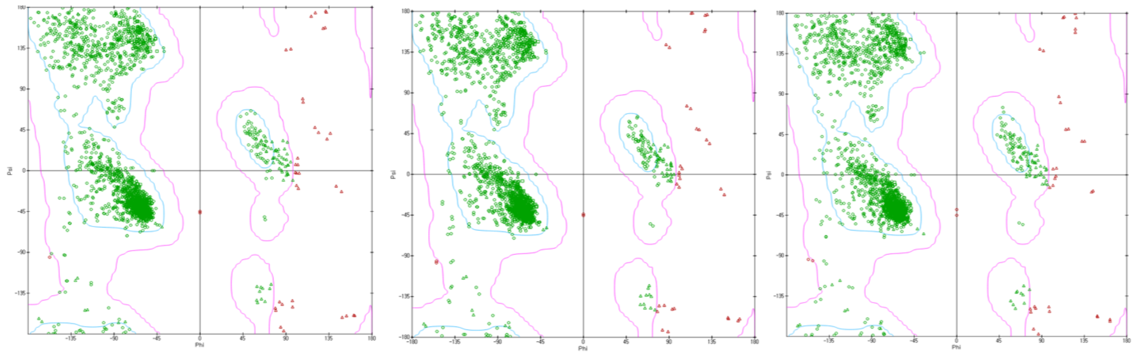

MCR From*M.thermautotrophicus*	Template structure (PDB 1HBM)
Favored	97.20 %
Outliers	0.16 %
Rotamer Outliner	1.66%
1HBM vs Query (RMSD)	0.59Å
Total structure weight	276.9kDa

MCR From*M.thermautotrophicus*	Template structure (PDB 1HBN)
Favored	97.37%
Outliers	0.08%
Rotamer Outliner	1.86%
1HBN vs Query (RMSD)	0.54Å
Total structure weight	277.25kDa

MCR From *M.marburgenesis Str. Marbug*	Template structure (PDB 3M32)
Favored	97.61%
Outliers	0.04%
Rotamer Outliner	1.47%
3M32 vs Query (RMSD)	0.54Å
Total structure weight	277.15kDa

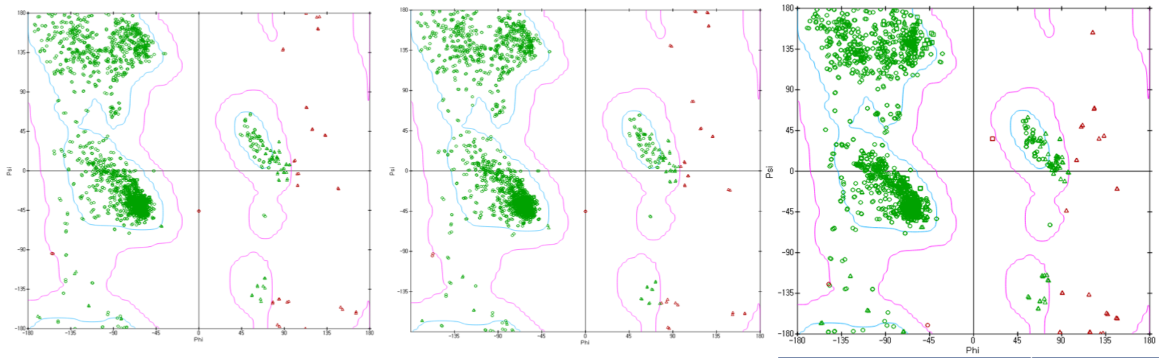

MCR From *M.marburgenesis Str. Marbug*	Template structure (PDB 5A0Y)
Favored	97.37%
Outliers	0.08%
Rotamer Outliner	0.58%
5A0Y vs Query (RMSD)	0.56Å
Total structure weight	276.76kDa

MCR From *M.marburgenesis Str. Marbug*	Template structure (PDB 5G0R)
Favored	97.53%
Outliers	0.08%
Rotamer Outliner	0.67%
5G0R vs Query (RMSD)	0.56Å
Total structure weight	276.58kDa

MCR From *Methanobrevibacter ruminantium*	Query structure
Favored	96.31%
Outliers	0.20%
Rotamer Outliner	0.20%
Total Residues	2,476
Total structure weight	269.9kDa

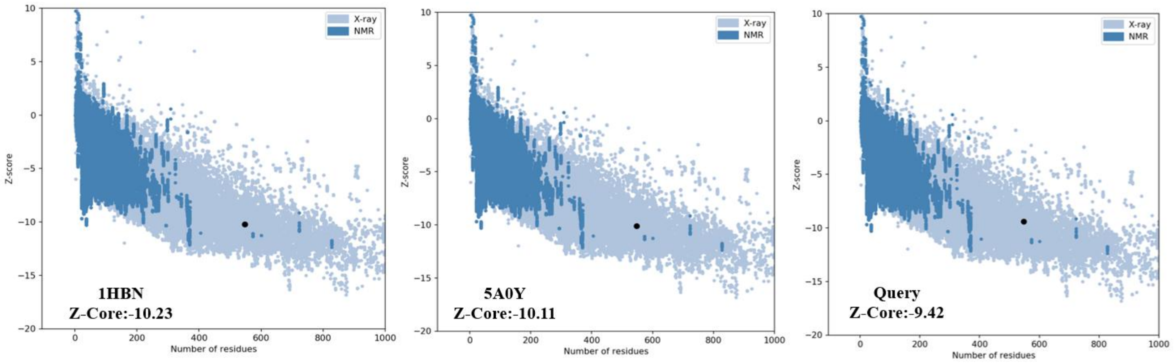

Figure S11. Stereochemical validation of Mcr structural models. Ramachandran plots and ProSA Z-scores are presented for the five template structures and the *M. ruminantium* Mcr query model**.** The query model exhibits high structural fidelity, with over 96% of residues residing in the most favored regions of the Ramachandran plot. The calculated Z-scores of -9.42 for the query model is situated within the central distribution of scores for experimentally determined protein structures of similar size, confirming the reliability and quality of the generated fold.

Table S1. Structural templates utilized for homology modeling of the *M. ruminantium* Mcr complex.

Template (PDB ID)	Crystal structure (Resolution Å)	Source Organism	Description/Active site ligandation
1HBU	1.90	*M. marburgenesis* str. *Marbug*	$Factor_{430}$, Coenzyme B, 1-thioethanesulfonic acid
**1HBN**	1.16	*M. thermautotrophicus*	$Factor_{430}$, Coenzyme B, 1-thioethanesulfonic acid
1HBO	1.78	*M. themautotrophicus*	$Factor_{430}$, Coenzyme B, 1-thioethanesulfornic acid
**1HBM**	1.80	*M. themautotrophicus*	$Factor_{430}$, O-phosphono-N-{(2E)-7-[(2-sulfoethyl)dithio] hept-2-enoyl}-L-threonine
5A8R	2.15	*M. marburgensis*	$Factor_{430}$, Coenzyme B, 1-thioethanesulfornic acid
**3M32**	1.35	*M. marburgenesis* str. *Marbug*	$Factor_{430}$, Coenzyme B, 1-thioethanesulfornic acid, O-phosphono-N-{(2E)-7-[(2-sulfoethyl)dithio] hept-2-enoyl}-L-threonine
3M30	1.45	*M. marburgenesis* str. *Marbug*	$Factor_{430}$, Coenzyme B, 1-thioethanesulfornic acid, O-phosphono-N-(9-sulfanylnonanoyl)-L-threonine
3M2V	1.80	*M. marburgenesis* str. *Marbug*	$Factor_{430}$, Coenzyme B, 1-thioethanesulfornic acid, O-phosphono-N-(8-sulfanyloctanoyl)-L-threonine
3M2R	1.30	*M. marburgenesis* str. *Marbug*	$Factor_{430}$, Coenzyme B, 1-thioethanesulfornic acid, O-phosphono-N-(5-sulfanylpentanoyl)-L-threonine
3M1V	1.45	*M.marburgenesis* str. *Marbug*	$Factor_{430}$, Coenzyme B, 1-thioethanesulfornic acid
3M2U	1.40	*M.marburgenesis* str. *Marbug*	$Factor_{430}$, Coenzyme B, 1-thioethanesulfornic acid, O-phosphono-N-(6-sulfanylhexanoyl)-L-threonine
**5A0Y**	1.10	*M.marburgenesis* str. *Marbug*	$Factor_{430}$, Coenzyme B, 1-thioethanesulfornic acid
7SXM	2.50	*M.marburgenesis* str. *Marbug*	$Factor_{430}$, Coenzyme B, 1-thioethanesulfornic acid
3POT	1.20	*M. marburgensis*	$Factor_{430}$, Coenzyme B, 1-thioethanesulfornic acid, O-phosphono-N-(6-sulfanylhexanoyl)-L-threonine
7B2H	2.12	*M. marburgenesis* str. *Marbug*	$Factor_{430}$, Coenzyme B, 1-thioethanesulfornic acid
**5G0R**	1.25	*M. marburgensis*	$Factor_{430}$, Coenzyme B

7SUC	1.90	*M. marburgenesis* str. *Marbug*	$Factor_{430}$, Coenzyme B, 1-thioethanesulfornic acid
1MRO	1.16	*M. marburgenesis* str. *Marbug*	$Factor_{430}$, Coenzyme B, 1-thioethanesulfornic acid

* Identification of high-resolution reference protein structure exhibiting near-complete sequence coverage (99%) to ensure high-fidelity architectural reconstruction of the Mcr model. The Mcr active site harbors the covalently linked CoM-S-S-CoB heterodisulfide in the $Mcr_{silient}$ State, whereas it accommodates discrete CoM-SH and CoB-SH cofactors in the $Mcr_{ox1\text{-}silent}$ State.

Table S2. Identification of key residues involved in the binding of cofactor F_{430} and co-substrates (CH_3-S-CoM, CoB-SH and CoM-S-S-CoB) across characterized Mcr states.

Active site	1HBM<br>(Mcr_{silent} from *M.thermautotrophicus*)	1HBN<br>($Mcr_{oxi-silent}$ from *M.thermautotrophicus*)	3M32<br>(Mcr_{silent} from *M.marburgenesis* str. *Marbug*)
**Mcrα**	Lys4, Lys11, Phe14, Pro58, Ile60, Thr62, Arg102, Ala144, Val145, Val146, Gln147, Asp170, Ala173, Asp174, Asp183, Asn185, Ser215, Arg216, C+ys218, Ser224, Arg225, Gln230, Met233, Ala243, Lys256, His262, Arg270, Glu275, Leu320, Met324, Ser325, Gly326, Gly327, Val328, Gly329, Phe330, Thr331, Gln332, Tyr333, Asp345, Tyr348, Glu352, Tyr380, Gly397, Gly442, Phe443, Met480, Val482, Val519, Lys531, Pro537, Glu538, Ala544, Tyr547, Pro548	Lys4, Lys11, Phe14, Pro58, Ile60, Thr62, Arg102, Glu117, Val124, Ala144, Val145, Val146, Gln147, Asp170, Ala173, Asp174, Asp183, Asn185, Ser215, Arg216, Cys218, Ser224, Arg225, Gln230, Met233, Ala243, Lys256, His262, Arg270, Glu275, Leu320, Met324, Gly326, Gly327, Val328, Gly329, Phe330, Thr331, Gln332, Tyr333, Asp345, Tyr348, Glu352, Tyr380, Phe396, Gly397, Gly442, Phe443, Tyr444, Met480, Asn481, Val482, Lys531, Phe537, Glu538, Ala544, Thr547, Thr547, Pro548	Arg102, Ala144, Val145, Val146, Gln147, Ser215, Arg216, Cys218, Arg225, Gln230, Met233, Ala243, Lys256, Arg270, Leu320, Met324, Ser325, Gly326, Gly327, Val328, Gly329, Phe330, Tyr331, Gln332, Tyr333, Tyr348, Phe396, Gly397, Gly442, Phe443, Tyr444, Met480, Asn481, Val482, Val519, Lys531, Phe537, Glu538
**Mcrβ**	Lys3, Glu25, Ser28, Arg31, Val95, Asp99, Thr101, Glu234, Arg235, Ser365, Ile366, Tyr367, Gly368, Gly369, His379, Ile380, Asn441	Glu25, Ser28, Leu30, Arg31, Val95, Asp99, Thr101, Glu234, Arg235, Asp271, Ala300, Phe361, Ser365, Ile366, Tyr367, Gly369, His379, Ile380, Asn441	Lys261, Asp271, Phe361, Phe362, Ser365, Ile366, Tyr367, Gly368, Gly369, His379, Ile380, Val381
**Mcrγ**	Glu30, Tyr109, Arg110, Leu117, Ser118, Gly119, Asp129, Lys153, Ser154, Val155, His156, His158	Glu30, Tyr109, Arg110, Leu117, Ser118, Gly119, Arg120, Asp129, Lys153, Ser154, Val155, His156, His158	Glu30, Arg43, Gly46, Glu47, Glu48, Tyr49, Leu117, Ser118, Gly119, Arg120, Lys153, Ser154, Val155, His156, His158
**Mcrα'**	Lys11, Phe14, Pro58, Ile60, Thr62, Arg102, Ala144, Val145, Val146, Gln147, Asp170, Ala173, Asp174, Asp183, Asn185, Ser215, Arg216, Cys218, Ser224, Arg225, Gln230, Met233, Ala243, Lys256, His262, Arg270, Glu275, Gln291, Arg294, Leu320, Met324, Gly326, Gly327, Val328, Gly329, Phe330, Thr331, Gln332, Tyr333, Asp345, Tyr348, Glu352, Tyr380, Gly397, Gly442, Phe443, Met480, Val482, Phe513, Asp515, Asp516, Phe520, Asp521, Phe522, Thr523	Lys11, Phe14, Pro58, Ile60, Thr62, Arg102, Ala144, Val145, Val146, Gln147, Asp170, Ala173, Asp174, Glu175, Asp183, Asn185, Ser215, Arg216, Cys218, Ser224, Arg225, Gln230, Met233, Ala243, Lys256, His262, Arg270, Glu275, Gln291, Arg294, Leu320, Met324, Gly326, Gly327, Val328, Gly329, Phe330, Thr331, Gln332, Tyr333, Asp345, Glu352, Asp356, Tyr380, Phe396, Gly397, Gly442, Phe443, Tyr444, Met480, Asn481, Val482, Phe513, Asp515, Phe520, Asp521, Phe522	Arg102, Ala144, Val145, Val146, Gln147, Ser215, Arg216, Cys218, Arg225, Gln230, Met233, Ala243, Lys256, Arg270, Leu320, Met324, Ser325, Gly326, Gly327, Val328, Gly329, Phe330, Tyr331, Gln332, Tyr333, Tyr348, Phe396, Gly397, Gly442, Phe443, Tyr444, Met480, Asn481, Val482
**Mcrβ'**	Lys3, Glu25, Ser28, Arg31, Val95, Glu234, Arg235, Ser365, Ile366, Tyr367, Gly369, His379, Ile380	Lys3, Glu25, Ser28, Leu30, Arg31, Val95, Asp147, Glu234, Arg235, Asp271, Phe361, Ser365, Ile366, Tyr367, Gly368, Gly369, His379, Ile380	Phe361, Phe362, Ser365, Ile366, Tyr367, Gly368, Gly369, His379, Ile380
**Mcrγ'**	Glu30, Leu117, Ser118, Gly119, Lys153, Ser154, Val155, His156, His158	Glu30, Glu34, Asp35, Lys38, Leu117, Ser118, Gly119, Arg120, Lys153, Ser154, Val155, His156, His158	Glu30, Arg43, Gly46, Glu47, Leu117, Ser118, Gly119, Arg120, Lys153, Ser154, Val155, His156, His158, Gln235, Arg236, Val239

Table S3. Computational alanine scanning of key interface in the *M. ruminantium* $Mcr_{oxi\text{-}silent}$ complex. Values represent the differences in total binding free energy ($\Delta\Delta G_{binding}$, kcal/mol) between wild-type and alanine-mutated structures at pH7.4. The ligand include cofactor and the co-substrates HS-CoM and CoB-SH on the key interface residues in **Fig. 5**.

Ni-F430 cofactor binding site in the $Mcr_{oxi\text{-}silent}$/all ligands complexes					
Mutation	Mutation energy[a]	Effect of mutation[b]	VDW term	Electrostatic term	Entropy term
A:Gly325Ala	9.01	Destabilizing	16.02	2.05	-0.03
A:Gly326Ala	1.24	Destabilizing	0.83	1.66	-0.01
A:Val327Ala	0.43	Neutral	3.47	-2.62	0.01
A:Gly328Ala	0.47	Neutral	-0.45	1.39	0.00
A:Phe329Ala	-2.38	Stabilizing	3.64	-9.85	0.91
A:Thr330Ala	-0.43	Neutral	1.11	-1.98	0.00
A:Gln331Ala	-0.47	Neutral	1.68	-2.62	0.00
A:Tyr332Ala	-2.04	Stabilizing	7.24	-11.36	0.02
A:Phe396Ala	-1.05	Stabilizing	2.49	-4.81	0.18
A:Gly397Ala	2.95	Destabilizing	2.80	3.14	-0.03
A:Gly398Ala	0.52	Destabilizing	0.44	0.58	0.01
A:Gln400Ala	-0.48	Neutral	1.76	-3.06	0.21
A:Gly442Ala	0.54	Destabilizing	-0.03	1.10	0.00
A:Phe443Ala	-3.67	Stabilizing	1.29	-9.91	0.80
B:Ser365Ala	-0.28	Neutral	0.13	-0.65	-0.02
B:Ile366Ala	-0.11	Neutral	2.69	-3.19	0.18
B:Tyr367Ala	-0.83	Stabilizing	7.37	-9.37	0.21
C:Leu117Ala	-0.31	Neutral	2.11	-2.82	0.06
C:Ser118Ala	0.09	Neutral	0.73	-0.54	-0.01
C:Gly119Ala	1.91	Destabilizing	1.26	2.59	-0.02
C:Arg120Ala	0.71	Destabilizing	3.33	-1.95	0.03
C:Ala153Ala	-0.01	Neutral	0.00	-0.02	0.00
C:Gly154Ala	0.08	Neutral	-0.19	0.34	0.00
C:Val155Ala	-0.84	Stabilizing	0.83	-2.55	0.03
C:His156Ala	-0.67	Stabilizing	0.90	-2.29	0.03
C:Gly157Ala	0.07	Neutral	-0.06	0.20	0.00
C:His158Ala	-0.73	Stabilizing	0.81	-2.27	0.00
C:Ser159Ala	-0.21	Neutral	0.09	-0.5	0.00
D:Ala144Ala	-0.01	Neutral	0.00	-0.02	0.00
D:Ala145Ala	-0.01	Neutral	0.00	-0.02	0.00
D:Val146Ala	0.00	Neutral	1.68	-1.83	0.09
D:Gln147Ala	-0.83	Stabilizing	5.62	-5.03	-1.41
D:Met150Ala	0.07	Neutral	4.84	-4.72	0.01
D:Val151Ala	-0.72	Stabilizing	0.47	-1.92	0.00
D:Met229Ala	-0.71	Stabilizing	0.44	-1.87	0.01
D:Gln230Ala	-0.79	Stabilizing	1.25	-2.98	0.09
D:Met233Ala	0.20	Neutral	3.57	-3.08	-0.06
D:Ile236Ala	-0.54	Stabilizing	0.50	-1.62	0.03
D:Ala243Ala	-0.01	Neutral	0.00	-0.03	0.00
D:Gly244Ala	0.14	Neutral	-0.72	1.01	-0.01

[a]The total binding energy function incorporates components: the van der Waals interaction energy, electrostatic interaction energy (calculated as a sum of coulombic interactions and polar solvation contributions), a side-chain conformational entropy term, and non-polar surface contribution to the mutation energy [60]. The non-polar surface-dependent term provides a consistent background contribution before and after the alanine mutation; its net contribution to differential mutation energy is negligible (0.00kcal/mol; data not shown in **Table S3**). [b]The energetic effect of each mutation is classified based on the resulting energy changes ($\Delta\Delta G$): stabilizing ($\Delta\Delta G$ < -0.5kcal/mol), natural (-0.5kcal/mol ≤ $\Delta\Delta G$ ≤ 0.5kcal/mol), and destabilizing ($\Delta\Delta G$ > 0.5kcal/mol).

Table S4. Computational alanine scanning of key interface residues in the *M. ruminantium* $Mcr_{oxi\text{-}silent}$ complex. Values represent the change in total binding free energy ($\Delta\Delta G_{binding}$, kcal/mol) between

wild-type and alanine-mutated structures at pH 7.4. The binding model incorporates the Ni-F_{430} cofactor and co-substrates HS-CoM and CoB-SH on the key interface residues in **Fig.5**.

Coenzyme CoM-SH binding site in the $Mcr_{oxi-silent}$/all ligands complexes					
Mutation	Mutation energy[a]	Effect of mutation[b]	VDW term	Electrostatic term	Entropy term
A:Phe329Ala	-2.38	Stabilizing	3.64	-9.85	0.91
A:Tyr332Ala	-2.04	Stabilizing	7.24	-11.36	0.02
A:Phe443Ala	-3.67	Stabilizing	1.29	-9.91	0.80
A:Tyr444Ala	-1.06	Stabilizing	0.56	-2.72	0.02
A:Gly445Ala	1.12	Destabilizing	0.49	1.76	0.00
B:Phe361Ala	-3.19	Stabilizing	2.94	-9.92	0.38
B:Ser365Ala	-0.28	Neutral	0.13	-0.65	-0.02
B:Tyr367Ala	-0.83	Stabilizing	7.37	-9.37	0.21
C:Leu117Ala	-0.31	Neutral	2.11	-2.82	0.06
C:Arg120Ala	0.71	Destabilizing	3.33	-1.95	0.03
Coenzyme CoB-SH binding site in the $Mcr_{oxi-silent}$/all ligands complexes					
A:Arg270Ala	1.08	Destabilizing	2.72	-2.00	0.90
A:Arg271Ala	0.53	Destabilizing	0.22	0.75	0.06
A:Leu319Ala	-0.35	Neutral	1.49	-2.29	0.06
A:Met323Ala	0.51	Destabilizing	2.73	-1.88	0.11
A:Ser324Ala	-0.03	Neutral	0.5	-0.55	0.00
A:Phe329Ala	-2.38	Stabilizing	3.64	-9.85	0.91
A:Phe443Ala	-3.67	Stabilizing	1.29	-9.91	0.80
A:Met480Ala	-0.51	Stabilizing	0.85	-1.86	0.00
A:Asn481Ala	-0.91	Stabilizing	0.44	-2.26	0.00
A:Val482Ala	-1.42	Stabilizing	1.51	-4.39	0.02
B:Phe361Ala	-3.19	Stabilizing	2.94	-9.92	0.38
B:Phe362Ala	-1.44	Stabilizing	3.05	-7.38	0.91
B:Tyr367Ala	-0.83	Stabilizing	7.37	-9.37	0.21
B:Gly368Ala	1.78	Destabilizing	2.46	1.15	-0.03
B:Gly369Ala	0.42	Neutral	-0.22	1.05	0.00
B:His379Ala	0.15	Neutral	0.18	-1.15	0.79
B:Val380Ala	-0.06	Neutral	0.99	-1.26	0.09
B:Val381Ala	-0.88	Stabilizing	0.5	-2.27	0.01
D:Arg225Ala	1.56	Destabilizing	1.58	1.18	0.22
D:Lys256Ala	3.54	Destabilizing	2.29	4.12	0.42
D:His257Ala	0.10	Neutral	0.93	-2.40	1.04

[a]The binding energy function incorporates several discrete components: van der Waals interactions, electrostatic energy (calculated as the sum of coulombic interactions and polar solvation contributions), a side-chain conformational entropy term, and a non-polar surface (surface tension) contribution to the mutation energy [60]. The non-polar surface-dependent term remained constant before and after alanine substitution; consequently, its net contribution to the differential mutation energy ($\Delta\Delta G$) was negligible (0.00kcal/mol; data not shown in **Table S4**).

[b]The energetic impact of each mutation was classified based on the $\Delta\Delta G$ threshold: stabilizing ($\Delta\Delta G$ < -0.5kcal/mol), natural (-0.5kcal/mol ≤ $\Delta\Delta G$ ≤ 0.5kcal/mol), and destabilizing ($\Delta\Delta G$ > 0.5kcal/mol).

Table S5. Computational alanine scanning of key interface residues in the *M. ruminantium* Mcr_{silent} compelx. Values represent the change in total binding free energy ($\Delta\Delta G_{binding}$, kcal/mol) between wild-type and alanine-mutated structures at pH 7.4. The binding model incorporates the Ni-F_{430} cofactor and the reaction product CoM-S-S-CoB. Positive values indicate a loss of binding affinity upon mutation on the key interface residues in **Fig.6**.

Ni-F430 cofactor binding site in the Mcr_{silent}/all ligands complexes					
Mutation	Mutation energy[a]	Effect of mutation[b]	VDW term	Electrostatic term	Entropy term
A:Gly325Ala	9.16	Destabilizing	15.75	2.63	-0.04
A:Gly326Ala	3.55	Destabilizing	4.45	2.67	-0.01
A:Val327Ala	0.22	Neutral	4.80	-3.82	-0.34
A:Gly328Ala	0.52	Destabilizing	-0.3	1.33	0.00
A:Phe329Ala	-6.21	Stabilizing	1.54	-14.76	0.50
A:Thr330Ala	-0.73	Stabilizing	0.86	-2.31	0.00
A:Gln331Ala	-0.16	Neutral	4.59	-4.67	-0.15
A:Tyr332Ala	-5.95	Stabilizing	0.76	-13.31	0.41
A:Phe396Ala	-0.92	Stabilizing	4.53	-6.80	0.27
A:Gly397Ala	1.03	Destabilizing	0.34	1.70	0.01
A:Gly398Ala	0.76	Destabilizing	0.95	0.56	0.01
A:Gln400Ala	-0.58	Stabilizing	1.30	-2.51	0.03
A:Gly442Ala	0.49	Neutral	-0.21	1.19	0.00
A:Phe443Ala	-4.52	Stabilizing	1.65	-11.32	0.39
B:Ser365Ala	-0.56	Stabilizing	-0.11	-0.98	-0.02
B:Ile366Ala	-0.45	Neutral	2.52	-3.71	0.18
B:Tyr367Ala	-1.81	Stabilizing	7.10	-11.34	0.39
C:Leu117Ala	-0.41	Neutral	1.26	-2.27	0.12
C:Ser118Ala	0.10	Neutral	0.32	-0.10	-0.01
C:Gly119Ala	1.15	Destabilizing	0.70	1.63	-0.02
C:Arg120Ala	0.28	Neutral	2.22	-1.68	0.01
C:Ala153Ala	0.03	Neutral	0.13	-0.08	0.01
C:Gly154Ala	0.30	Neutral	0.05	0.55	0.00
C:Val155Ala	-0.77	Stabilizing	0.81	-2.39	0.03
C:His156Ala	-0.49	Neutral	1.08	-2.07	0.00
C:Gly157Ala	0.03	Neutral	-0.08	0.15	0.00
C:His158Ala	-0.46	Neutral	1.30	-2.23	0.01
C:Ser159Ala	-0.14	Neutral	0.09	-0.37	0.00
D:Ala144Ala	-0.01	Neutral	0.01	-0.03	0.00
D:Ala145Ala	-0.04	Neutral	0.18	-0.28	0.01
D:Val146Ala	0.16	Neutral	1.81	-1.64	0.09
D:Gln147Ala	-0.34	Neutral	6.48	-4.87	-1.43
D:Met150Ala	-0.05	Neutral	3.67	-4.29	0.32
D:Val151Ala	-0.47	Neutral	0.77	-1.72	0.01
D:Met229Ala	-0.86	Stabilizing	0.35	-2.08	0.01
D:Gln230Ala	-1.06	Stabilizing	1.32	-3.59	0.09
D:Met233Ala	0.32	Neutral	3.99	-3.28	-0.04
D:Ile236Ala	-0.32	Neutral	0.58	-1.27	0.03
D:Gly244Ala	0.84	Destabilizing	0.23	1.48	-0.02

[a]The binding free energy function incorporates several discrete components: van der Waals interactions, electrostatic energy (calculated as the sum of coulombic interactions, electrostatic energy (calculated as the sum of coulombic interactions and polar solvation contributions), a side-chain conformational entropy term, and a non-polar surface (surface tension) contribution to the mutation energy [60]. The non-polar surface-dependent mutation energy ($\Delta\Delta G$) was negligible (0.00kcal/mol; data not shown in **Table S5**).

[b]The energetic impact of each mutation was classified based on the $\Delta\Delta G$ threshold: stabilizing ($\Delta\Delta G$ < -0.5kcal/mol), natural (-0.5kcal/mol ≤ $\Delta\Delta G$ ≤ 0.5kcal/mol), and destabilizing ($\Delta\Delta G$ > 0.5kcal/mol).

Table S6. Computational alanine scanning of key interface residues in the *M. ruminantium* Mcr_{silent} complex. Values represent the change in total binding free energy ($\Delta\Delta G_{binding}$, kcal/mol) between

wild-type and alanine-mutated structures at pH 7.4. The binding model incorporates the Ni-F_{430} cofactor and the reaction product CoM-S-S-CoB. Positive values indicate a loss of binding affinity upon mutation on the key interface residues in **Fig.6**.

Heterodisulfide CoM-S-S-CoB binding site in the Mcr_{silent}/all ligands complexes					
Mutation	Mutation energy[a]	Effect of mutation[b]	VDW term	Electrostatic term	Entropy term
A:Arg270Ala	2.09	Destabilizing	2.80	0.10	0.80
A:Arg271Ala	0.11	Neutral	0.27	-0.14	0.06
A:Leu319Ala	-0.61	Stabilizing	1.44	-2.74	0.05
A:Met323Ala	1.03	Destabilizing	2.77	-0.89	0.11
A:Ser324Ala	-0.23	Neutral	0.55	-1.01	0.00
A:Phe329Ala	-3.60	Stabilizing	4.64	-12.87	0.64
A:Tyr332Ala	-1.79	Stabilizing	8.61	-12.81	0.64
A:Phe443Ala	-4.52	Stabilizing	1.65	-11.32	0.39
A:Tyr444Ala	-1.17	Stabilizing	0.22	-2.59	0.02
A:Met480Ala	-0.30	Neutral	0.83	-1.45	0.01
A:Asn481Ala	-0.88	Stabilizing	0.38	-2.13	0.00
A:Val482Ala	-2.64	Stabilizing	0.54	-5.84	0.01
B:Phe361Ala	-2.82	Stabilizing	3.17	-9.85	0.65
B:Phe362Ala	-0.95	Stabilizing	3.20	-6.32	0.76
B:Tyr367Ala	-1.81	Stabilizing	7.10	-11.34	0.39
B:Gly368Ala	0.73	Destabilizing	1.69	-0.19	-0.03
B:Gly369Ala	0.24	Neutral	-0.26	0.75	0.00
B:His379Ala	-0.05	Neutral	0.52	-1.67	0.65
B:Val380Ala	0.36	Neutral	1.35	-0.78	0.09
B:Val381Ala	-0.47	Neutral	0.58	-1.53	0.01
D:Arg225Ala	1.26	Destabilizing	1.99	0.28	0.16
D:Lys256Ala	2.72	Destabilizing	2.56	2.42	0.29
D:His257Ala	0.29	Neutral	0.96	-1.99	1.00

[a]The binding free energy function incorporate several discrete components: van der Waals interactions, electrostatic energy (calculated as the sum of coulombic interactions and polar solvation contributions), a side-chain conformational entropy term, and a non-polar surface (surface tension) contribution to the mutation energy. The non-polar surface-dependent term provided a constant background contribution to the differential mutation energy ($\Delta\Delta G$) was negligible (0.00kcal/mol; data not shown in **Table S6**).

[b]The energetic impact of each mutation was classified based on the $\Delta\Delta G$ threshold: stabilizing ($\Delta\Delta G$ < -0.5kcal/mol), natural (-0.5kcal/mol ≤ $\Delta\Delta G$ ≤ 0.5kcal/mol), and destabilizing ($\Delta\Delta G$ > 0.5kcal/mol).
